# RNA‐Binding Proteins: Function, Biological Mechanisms, and Therapeutic Opportunities

**DOI:** 10.1002/mco2.70938

**Published:** 2026-08-24

**Authors:** Ling Li, Xiuli Yan, Qing Ji, Hui Zhang

**Affiliations:** ^1^ Institute of Interdisciplinary Integrative Medicine Research Shanghai University of Traditional Chinese Medicine Shanghai China; ^2^ Yueyang Hospital of Integrated Traditional Chinese and Western Medicine Shanghai University of Traditional Chinese Medicine Shanghai China; ^3^ Department of Medical Oncology & Cancer Institute of Integrative Medicine Shuguang Hospital, Shanghai University of Traditional Chinese Medicine Shanghai China

**Keywords:** post‐transcriptional gene regulation, precision oncology, RBP interactome, regulatory networks, RNA‐binding proteins, targeted therapy

## Abstract

RNA‐binding proteins (RBPs) are central regulators of post‑transcriptional gene expression, controlling RNA stability, localization, translation, and alternative splicing. Their functions arise not only from intrinsic RNA‐binding domains but also from dynamic interactions with noncoding RNAs, metabolites, cofactors, and other RBPs. Here, we summarize the structural diversity and core biological activities of canonical and noncanonical RBPs, and delineate how competitive and cooperative regulatory networks dictate RBP function in disease, with an emphasis on cancer. Competitive mechanisms, including lncRNA‐mediated sequestration, antagonistic crosstalk between miRNAs and RBPs, and competition among RBPs for shared substrates, can redirect RNA fate. In contrast, cooperative mechanisms assemble multimolecular ribonucleoprotein complexes that reinforce oncogenic or tumor‐suppressive programs. Dysregulation of these networks promotes proliferation, metastasis, immune evasion, and therapy resistance. We also review emerging therapeutic strategies that target RBP‐centered regulatory circuits, including antisense oligonucleotides, small molecules, protein degraders, and natural products, and we evaluate representative preclinical studies and clinical trials. By integrating mechanistic principles with translational evidence, this review provides a network‐based framework for exploiting RBPs as therapeutic vulnerabilities and for advancing next‐generation precision oncology.

## Graphical abstract

RNA‐binding proteins (RBPs) regulate RNA stability, localization, translation, and splicing through intrinsic binding domains and interactions with diverse cellular partners. Their competitive and cooperative networks shape disease‐related RNA programs, especially in cancer. Dysregulated RBP activity drives tumor progression yet also creates therapeutic opportunities, including antisense oligonucleotides, small molecules, protein degraders, and natural products.

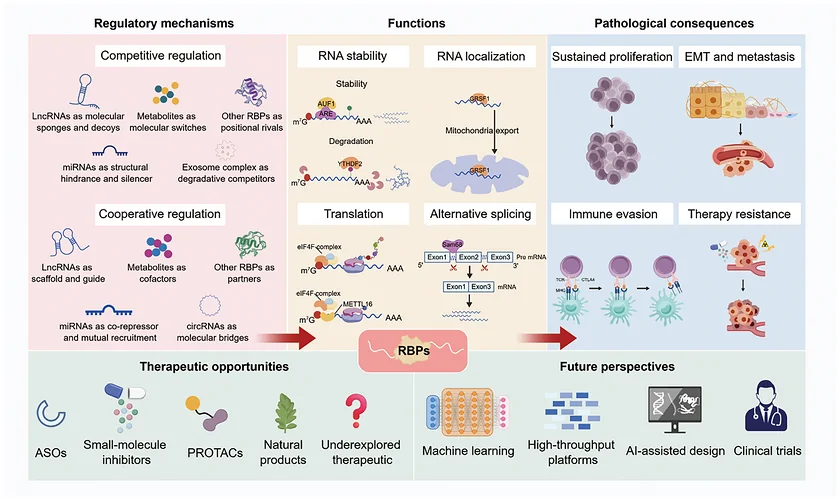

## 1 Introduction

RNA‐binding proteins (RBPs) constitute a large and conserved class of regulators in human cells, with approximately 1600 RBPs identified to date, accounting for about 7.5% of all protein‐coding genes [1, 2]. Through direct or indirect interactions with coding and noncoding RNAs, RBPs coordinate gene‐expression programs at multiple post‐transcriptional levels and thereby contribute to cellular homeostasis. They participate in a wide range of fundamental biological processes, including cell growth, development, differentiation, metabolism, and stress responses [3, 4, 5]. Functionally, RBPs are involved in virtually every step of post‐transcriptional regulation, from pre‐mRNA splicing and RNA processing to mRNA stability, translation, decay, and subcellular transport, thereby tightly supervising transcript formation and function to preserve cellular homeostasis [6, 7, 8].

Recent studies have further revealed that RBPs are key regulators of RNA modifications and higher order ribonucleoprotein assemblies, such as stress granules and phase‐separated condensates, adding an additional layer of control to the post‐transcriptional regulatory landscape [9, 10, 11]. Consistent with these diverse functions, RBPs can exert either protective or pathogenic effects depending on cellular context, disease state, and interacting partners. For example, several heterogeneous nuclear ribonucleoprotein (HNRNP) family members can promote aberrant splicing and enhance the stability or translation of disease‑associated transcripts, whereas protective RBPs such as KH domain containing RNA binding (QKI) often stabilize mRNAs that maintain cellular homeostasis and restrain pathological remodeling [12, 13, 14, 15].

Dysregulated RBP expression, localization, modification, or activity has therefore been implicated in a broad spectrum of human diseases, including cancer, neurodegenerative disorders, metabolic diseases, immune dysfunction, and cardiovascular disorders [16]. Among these, cancer represents one of the most extensively studied and therapeutically relevant disease contexts, in which RBPs remodel oncogenic and tumor‐suppressive transcriptomes to influence malignant progression.

Mechanistically, RBPs rarely act in isolation. High‐throughput approaches such as crosslinking and immunoprecipitation sequencing (CLIP‐seq) and systematic evolution of ligands by exponential enrichment (SELEX) have revealed that RBPs commonly function in combinatorial and context‐dependent networks, coordinated by their recognition of conserved RNA regulatory elements within shared targets [17, 18, 19]. Within these networks, RBPs engage in competitive and cooperative interactions with other RBPs, noncoding RNAs, and signaling molecules. In cancer, their dysregulation contributes to major malignant phenotypes, including sustained proliferation, metabolic reprogramming, epithelial–mesenchymal transition (EMT), immune evasion, and therapy resistance [20, 21, 22]. Therefore, elucidating how RBP activity is regulated by intrinsic structural features and extrinsic molecular partners is essential for understanding post‐transcriptional gene regulation in disease and for identifying new therapeutic vulnerabilities.

Despite the rapid expansion of RBP research, a systematic framework that connects RBP structural diversity, core RNA‐metabolic functions, dynamic interactome‐level mechanisms and therapeutic translation remains incomplete. In this review, we provide a comprehensive overview of current advances in RBP biology under the framework of function, biological mechanisms, and therapeutic opportunities. We first summarize the structural diversity of canonical and noncanonical RBPs and their fundamental roles in RNA metabolism. We then discuss how competitive and cooperative mechanisms involving noncoding RNAs, metabolites, cofactors, and other RBPs shape RBP‐centered regulatory networks. With cancer as a major focus, we further examine how dysregulation of these networks drives proliferation, EMT and metastasis, immune evasion, and therapy resistance, while also highlighting selected examples from other diseases to illustrate broader biological principles. Finally, we review emerging therapeutic strategies targeting RBPs and their regulatory interfaces, including antisense oligonucleotides (ASOs), small‐molecule inhibitors, targeted protein degradation technologies, and natural products, and critically evaluate representative preclinical and clinical studies of RBP‐centered interventions. By integrating mechanistic insights with translational advances, this review aims to provide a network‐based framework for understanding RBP function and for exploiting RBP‐associated vulnerabilities in precision medicine. Details of the search strategy and selection criteria are provided in the Supporting Information.

## 2 Structural Diversity and Fundamental Functions of RBPs

To understand how RBPs exert their diverse regulatory roles, it is essential to first understand their structural organization and domain composition. RBPs encompass a broad spectrum of canonical and noncanonical proteins characterized by distinct RNA‑binding motifs, many of which determine their binding specificity, subcellular distribution, and biochemical activities [23]. These structural features fundamentally shape how RBPs engage with RNA substrates and assemble into regulatory complexes. Therefore, this section begins by outlining the major classifications and structural motifs of RBPs, followed by an overview of their core functions across RNA metabolism.

### 2.1 Classification and Structural Motifs of RBPs

**FIGURE 1:**
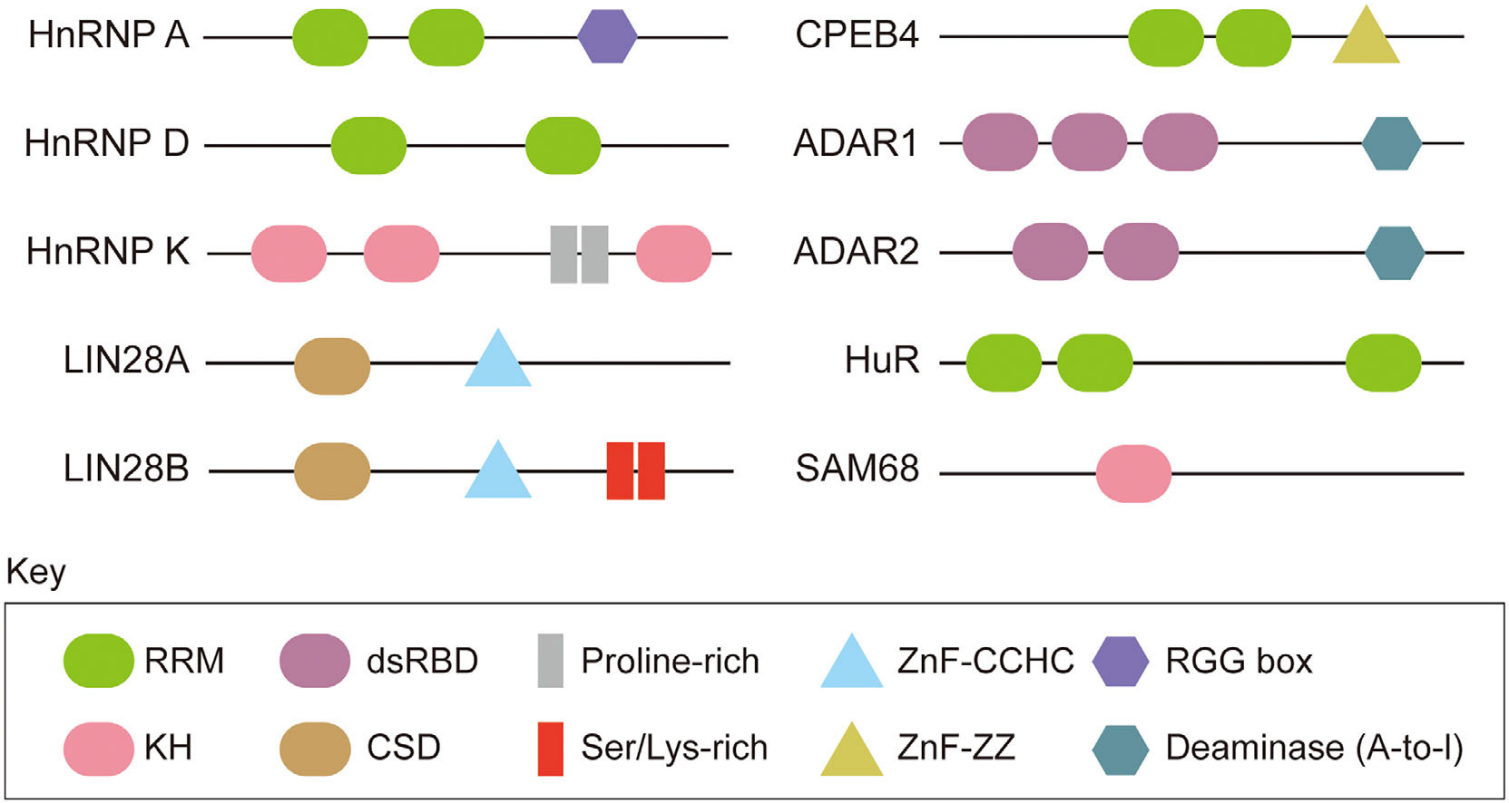
Common domains of RNA‑binding proteins (RBPs). This figure highlights representative domains frequently found in RBPs. Distinct colored boxes denote major domain types, including the RNA recognition motif (RRM), K homology (KH) domain, double‑stranded RNA‑binding domain (dsRBD), proline‑rich regions, serine/lysine‑rich segments, arginine–glycine–glycine (RGG) motifs, cold‑shock domains (CSDs), CCHC‐ and ZZ‑type zinc fingers, and deaminase domains involved in A‑to‑I RNA editing.

RBPs are structurally heterogeneous and are commonly classified as canonical or noncanonical according to whether they contain classical RNA‐binding domains (RBDs) [24]. Canonical RBPs typically harbor well‐defined motifs such as the RNA recognition motif (RRM), K homology (KH) domain, double‐stranded RNA‐binding domain (dsRBD), cold‐shock domain (CSD), zinc‐finger modules, and RGG/RG‐rich regions (Figure 1). Representative canonical RBPs include members of the HNRNP, LIN28, HuR, and ADAR families [25, 26, 27]. Many RBPs recognize sequence‐specific motifs, RNA secondary structures, or both through unique modular arrangements of these domains. In this way, using several RBDs in different combinations allows an RBP to recognize many more RNA targets and achieve higher binding specificity and affinity [28].

By contrast, a substantial fraction of RBPs lacks obvious classical RBDs but can still associate with RNA. Rather than reflecting true nonspecificity, this behavior is often context dependent and influenced by protein abundance, RNA availability, binding and dissociation kinetics, subcellular localization, and cooperation with cofactors [29]. In addition, many RBPs are enriched in intrinsically disordered regions (IDRs), which provide conformational flexibility and support multivalent RNA–protein and protein–protein interactions [30, 31]. Together, these structural features enable RBPs to function as hubs within ribonucleoprotein networks and form the molecular basis for their diverse roles in RNA metabolism.

### 2.2 Roles of RBPs in RNA Metabolism

**FIGURE 2:**
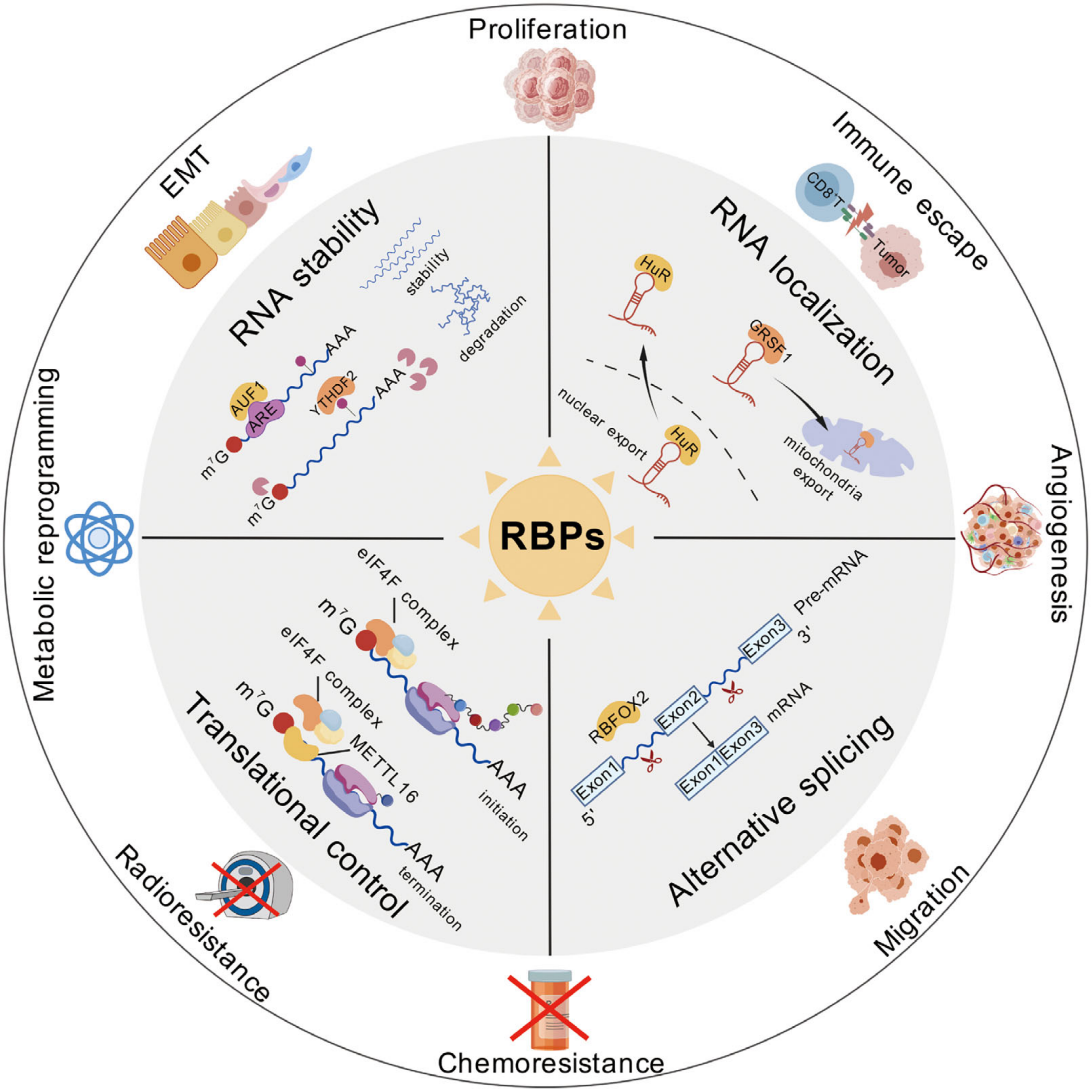
RBPs modulate the malignant progression of cancers. By regulating RNA stability, localization, splicing, and translation, RBPs impact key cancer phenotypes including proliferation, immune evasion, angiogenesis, migration, chemoresistance, radioresistance, metabolic reprogramming, and epithelial–mesenchymal transition (EMT).

RBPs are key regulators of post‐transcriptional gene expression and shape RNA fate through effects on stability, localization, translation, and alternative splicing. In cancer, these activities are frequently rewired to alter oncogene and tumor suppressor expression and promote malignant progression (Figure 2). The following subsections outline the key functional roles of RBPs in RNA metabolism.

#### 2.2.1 RNA Stability and Decay

The stability of eukaryotic mRNA is principally governed by structural elements at its termini, notably the 5′ m^7^G cap and the 3′ poly(A) tail, which cooperatively control transcript longevity and degradation kinetics [32, 33]. Two distinct enzymatic pathways mediate mRNA turnover in cytoplasmic compartments. The first cascade commences with progressive shortening of the poly(A) tail (deadenylation), triggering subsequent removal of the 5′ cap structure. This permits processive exonucleolytic digestion in the 5′–3′ orientation by 5′–3′ Exoribonuclease 1 (XRN1) and associated factors [34, 35]. Conversely, the alternative pathway involves poly(A) tail removal followed by 3′–5′ directional degradation catalyzed by the exosome complex and its cofactors [36, 37].

Growing evidence indicates that RBPs play critical roles in cancer progression by dynamically regulating the stability of specific target mRNAs [38, 39]. Mechanistically, RBPs orchestrate RNA fate through two opposing pathways. On the one hand, RBPs stabilize transcripts via sequence‐specific interactions with cognate RNA motifs [40]. Elevated AU‐rich RNA‐binding factor 1 (AUF1) expression, for example, drives colorectal cancer (CRC) metastasis by binding and stabilizing mRNAs encoding key signaling components such as pyruvate dehydrogenase kinase (PDK) and EMT transcription factors snail family zinc finger 1 (SNAI1) and twist family BHLH transcription factor 1 (TWIST1). Depleting AUF1 not only reduces the stability of these transcripts but also attenuates ERK and AKT pathway activation, underscoring its role in maintaining an aggressive tumor phenotype [41, 42]. Similarly, insulin‐like growth factor 2 mRNA‐binding protein 2 (IGF2BP2) supports tumor‐suppressive functions in clear cell renal cell carcinoma (ccRCC) by binding CKB mRNA and enhancing its stability, thereby inhibiting metastatic dissemination in vitro and in vivo [43, 44]. Besides, FUS binds near the 3′ end of GluA1 mRNA to maintain its poly(A) tail and stability, and loss of this regulation impairs synaptic function and contributes to frontotemporal dementia (FTLD)‑like pathophysiology [45].

On the other hand, RBPs also function as degradation catalysts by recognizing epitranscriptomic marks such as N6‐methyladenosine (m6A) [46]. Aberrant m6A reader protein expression drives RNA processing imbalance in malignancies, disrupting transcript stabilization fidelity and inducing aberrant translation of m6A‐modified mRNAs [47, 48]. Under hypoxic conditions in hepatocellular carcinoma (HCC), suppression of the m6A reader YTH domain‐containing family protein 2 (YTHDF2) leads to impaired degradation of EGFR transcripts, facilitating tumor growth; restoring YTHDF2 expression reverses this stabilization and suppresses tumorigenesis [49]. Besides, in glioblastoma, hyperactivated EGFR/SRC/ERK signaling enhances YTHDF2 stability, which in turn promotes degradation of cholesterol homeostasis genes such as liver X receptor alpha (LXRA) and human immunodeficiency virus Type I enhancer binding protein 2 (HIVEP2), driving metabolic adaptation and invasive growth [50]. RBPs exhibit dual regulatory capacity, not only stabilizing transcripts through sequence‐specific interactions but also directing degradation via recognition of epitranscriptomic modifications, thereby precisely modulating RNA homeostasis [51, 52]. Such regulatory versatility not only underscores their central role in cancer progression but also complicates therapeutic targeting, as the ultimate stability of an mRNA is often not determined by a single RBP, but by the dynamic equilibrium between competing stabilizing and destabilizing forces.

#### 2.2.2 Subcellular Localization and Transport

While most mRNAs are exported to the cytoplasm for translation, a select subset achieves precise subcellular localization—a process conferring spatial control over protein synthesis that proves essential for cellular polarization, migration, and local signaling [53, 54]. This compartment‐specific targeting is largely directed by RBPs, which recognize cis‐regulatory elements or structural motifs within 3′ UTRs to partition transcripts into distinct subcellular niches [55, 56]. For example, the cytotoxic granule‐associated RNA‐binding protein 1 (TIA1) nucleates stress granules by sequestering stress‐response transcripts like p53 mRNA, suppressing their protein synthesis through translational repression. DNA damage triggers TIA1 condensate disassembly, liberating p53 mRNA for polysomal recruitment, relocation, and translation during B lymphocyte activation [57]. Accumulating evidence has shown that TIA1 interacts with cancer‐related mRNA and play an important role in driving malignant progression [58, 59, 60]. Besides, RBPs human antigen R (HuR) and G‐rich sequence factor 1 (GRSF1) were reported to bind the lncRNA RMRP and facilitate its export from the nucleus. They then direct its specific localization, with HuR recruiting it to the cytosol and GRSF1 to the mitochondrial matrix [61].

Nagaoka et al. confirmed that cytoplasmic polyadenylation element‐binding protein 1 (CPEB1) governs the polarized localization of zonula occludens protein 1 (ZO‐1) mRNA in mammary epithelia, enabling apical assembly of junctional complexes. Disruption of this regulatory axis causes spatial uncoupling of ZO‐1 from syntaxin‐3 and aberrant partitioning of E‐cadherin, eroding epithelial polarity and leading to abnormal breast development [62]. Notably, IGF2BP1 associates with β‐actin mRNA in the nucleus [63]. Cytoplasmic translocation triggers mRNA translational suppression without compromising stability [64]. Following peripheral mRNA trafficking, Src‐mediated tyrosine phosphorylation at KH domains 2–3 interfaces induce mRNP complex dissociation, enabling ACTB translation and β‐actin polymerization [65, 66]. While current studies clearly show that specific RBPs can direct subcellular mRNA localization and influence cell polarity or migration, most evidence comes from a few well‑studied RBPs and limited cell models. Genome‑wide localization maps in solid tumors remain scarce, and it is still uncertain how well the existing paradigms generalize across cancer types. In addition, many findings rely on overexpression or short‑term knockdown, which may not reflect physiological RBP levels in patients. More systematic spatial and imaging‑based approaches will be needed to define how RBP‑mediated RNA trafficking shapes tumor heterogeneity and metastasis.

#### 2.2.3 Translation Regulation

The translation of mRNA is primarily regulated at the initiation stage [67, 68]. During this step, RBPs modulate translational efficiency by associating with specific motifs in the 5′ or 3′ UTRs of target mRNAs, thereby exerting either enhancing or repressive effects on protein synthesis [69, 70, 71]. The eukaryotic translation initiation factor 4F (eIF4F) is a protein complex that controls mRNA translation by recognizing the 5′ cap structure [72]. Dysregulated expression of eIF4E, a key subunit of the eIF4F complex, has been linked to nearly 30% of all human cancers [73]. Recent researches have shown that eIF4E knockdown mice not only maintain normal physiological functions but also exhibit significant suppression of tumor growth [74, 75]. Phosphorylation of eIF4E has been demonstrated to promote mRNA translation, which is essential for various cancer development [76, 77, 78, 79]. Moreover, treatment with eFT508, a specific inhibitor of phosphorylated eIF4E, disrupts the ability of cancer cells to translate specific metabolic genes under a ketogenic diet. This deprivation of alternative energy sources, such as ketone bodies, ultimately inhibits the growth of pancreatic cancer [80].

Beyond the 5′ UTR, 3′ UTRs serve a critical function in translational regulation [81]. For example, by binding to nucleotides 1403–1409 in the 3′ UTR of high mobility group box 1 (HMGB1), Musashi‐2 (MSI2) increases disulfide HMGB1 translation and fosters an immunosuppressive tumor microenvironment (TME) in CRC [82]. In addition, MSI2 enhances proliferation and chemoresistance in T‐cell acute lymphoblastic leukemia (T‐ALL) through the post‐transcriptional regulation of MYC [83]. Besides, MSI1 has been shown to promote MMP9‐mediated breast cancer (BC) metastasis by binding to the 3′ UTR of tissue inhibitor of metalloproteinases 3 (Timp3) mRNA and inhibiting its translation [84], illustrating that translational output is frequently an integrated outcome of multiple RBPs, which can act synergistically or be antagonized by competitors. The oncogenic role of translation factors such as eIF4E is strongly supported by genetic models and pharmacological inhibition, providing clear causal evidence. By contrast, most studies of RBPs acting on 5′ or 3′ UTRs rely on reporter assays and short‐term perturbations, which demonstrate transcript‑specific regulation but offer limited insight into their global translational impact in vivo. Moreover, many mechanisms have been defined in single tumor models, so their generalizability remains uncertain. Given the essential nature of cap‑dependent translation in normal tissues, it will be critical to identify context‑specific dependencies and therapeutic windows that enable selective targeting of malignant translation with acceptable toxicity.

#### 2.2.4 Alternative Splicing

Alternative splicing is a key mechanism that alters gene‐expression patterns across physiological and disease conditions, including cancer, neurodegeneration, metabolic disorders, immune dysfunction, and cardiovascular disease [85, 86]. Major types of aberrant splicing include constitutive splicing changes, exon skipping (ES)/inclusion, alternative 5′ or 3′ splice site selection, intron retention, and mutually exclusive exons [87, 88]. Among these, ES represents one of the most prevalent events regulated by RBPs [89]. For instance, RNA‐binding fox‐1 homolog 2 (RBFOX2) promotes the migration of pancreatic cancer cells by enhancing the skipping of exons in transcripts that encode proteins involved in cytoskeletal remodeling [90]. Additionally, karyopherin subunit alpha 2 (KPNA2) has been shown to regulate ES events, thereby facilitating the proliferation, migration, and invasion of gastric cancer (GC) cells [91], while loss of HuR triggers exon 11 skipping in Eif4enif1, generating the stable isoform 4E‐Ts, which enhances processing body assembly, accelerates decay of proangiogenic mRNAs, and suppresses pathological angiogenesis [92].

QKI, a splicing regulator which is often downregulated in numerous cancers, correlates with adverse clinical outcomes [93, 94, 95]. Zhao et al. have demonstrated that in papillary thyroid carcinoma, downregulation of QKI promotes metastasis by inducing exon 14 retention in extended synaptotagmin 2 (E‐Syt2), leading to the expression of a long isoform transcript (termed E‐Syt2L) [96]. Besides, the signal‐dependent phosphorylation of Sam68 regulates the inclusion of exon V5 sequences in CD44 mRNA, thereby generating a proinvasive cell adhesion protein variant [97]. Furthermore, threonine phosphorylation of Sam68 modulates the alternative splicing of Bcl‐x pre‐mRNA, leading to altered production of its proapoptotic (Bcl‐xS) or antiapoptotic (Bcl‐xL) isoforms [98].

Beyond malignancy, aberrant RBP‐mediated splicing also contributes to non‐neoplastic diseases. In metabolic disease contexts, altered Muscleblind‐like 1 (MBNL1)‐dependent splicing of the insulin receptor (INSR) exon 11 has been linked to insulin resistance and impaired glucose homeostasis [99]. In immune regulation, TRA2β‐dependent alternative splicing of an ultraconserved poison exon within its own transcript fine‑tunes TCR signaling thresholds, and its dysregulation disrupts the balance between T‑cell activation and survival [100]. In the cardiovascular system, RNA‐binding motif protein 20 (RBM20) controls titin (TTN) splicing, and RBM20 dysfunction causes dilated cardiomyopathy by producing maladaptive cardiac isoforms [101].

Collectively, these examples illustrate that RBPs can exert profound effects on disease‑associated splicing programs across cancer and other pathological conditions. However, much of the current evidence remains correlative rather than causal. Many splicing changes are inferred from bulk RNA‑seq expression patterns without definitive proof of direct RBP binding or functional necessity. Even when CLIP‑seq, minigene assays, or loss‑of‑function studies demonstrate direct regulation, most analyses focus on a limited number of exons, leaving the architecture of broader splicing networks poorly defined. Moreover, RBPs such as CPEB2 and QKI exhibit tumor‑promoting roles in some contexts but tumor‑suppressive functions in others, underscoring unresolved context‑dependent regulation and the need to understand how lineage‑specific and microenvironmental factors reshape their splicing outputs. More systematic, isoform‑resolved analyses in patient samples and in vivo disease models will be essential to map these networks comprehensively and to identify splicing regulators that can be safely and effectively targeted.

**FIGURE 3:**
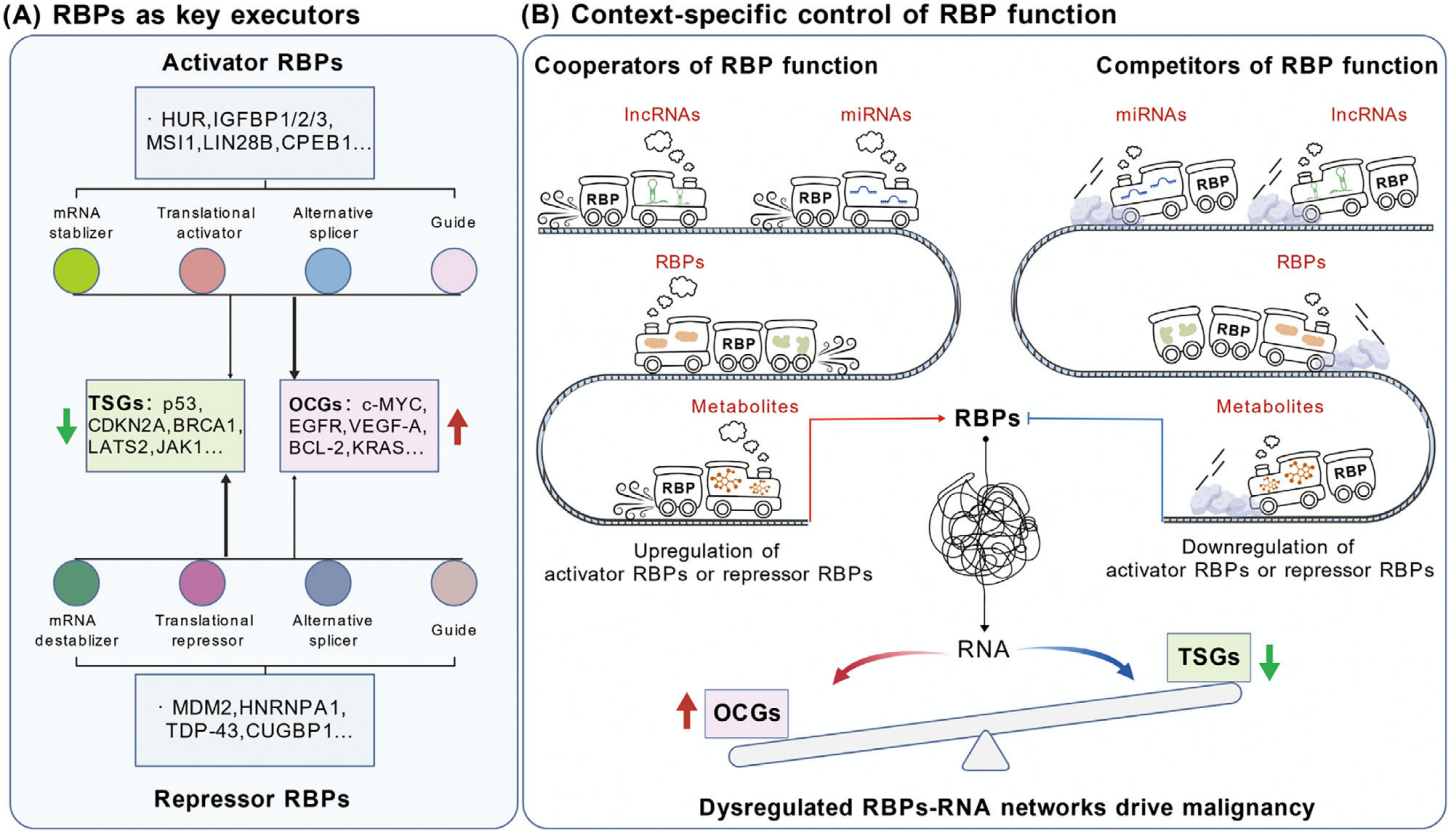
Oncogenic rewiring of RBP–RNA networks by competitive and cooperative partners. (A) RBPs as key executors. RBPs function broadly as activators or repressors. Activator RBPs promote oncogene (OCG) expression by stabilizing mRNAs, enhancing translation, or regulating alternative splicing, whereas repressor RBPs predominantly suppress tumor suppressor gene (TSG) expression through opposite mechanisms. Together, these opposing activities shape post‑transcriptional programs that support tumorigenesis. (B) Context‑dependent regulation: “train‑track” dynamics of competitors and collaborators. The oncogenic or tumor‑suppressive output of an RBP is dynamically modulated by upstream competitors and collaborators, including lncRNAs, miRNAs, metabolites, and other RBPs. This process resembles a “train system,” in which competitors act like underpowered engines that stall “RBP carriages” on rugged antitumor tracks, weakening tumor‑suppressive RBP activity and limiting TSG expression. In contrast, collaborators behave as fully powered locomotives that propel “RBP carriages” along high‑speed protumor tracks, amplifying oncogenic RBP functions and driving OCG expression. The imbalance between competitive and cooperative influences disrupts RBP–RNA network homeostasis and promotes tumorigenesis through coordinated dysregulation of TSG‑ and OCG‑associated pathways.

So far, we have outlined how RBPs regulate post‐transcriptional gene expression in cancer, highlighting their central role in shaping RNA fate. However, describing the functions of RBPs at their RNA targets provides only part of the pattern. A single RBP can regulate multiple transcripts, whereas several RBPs may cooperate or compete on the same RNA molecule [6, 102]. These interactions fine‐tune gene‐expression programs and contribute to cancer progression. Therefore, RBP activity should not be viewed as a fixed or purely cell‐autonomous property determined only by expression levels. Instead, it is dynamically shaped by the cellular context, including the TME and the molecular partners with which RBPs interact. In the following sections, we shift our perspective from viewing RBPs as solitary executors to understanding them as central nodes within a vast interactome. We focus on the competitive and cooperative mechanisms that tune RBP activity, and further discuss how dysregulation of these networks drives malignant phenotypes (Figure 3).

## 3 Biological Mechanisms of RBP Regulation and Dysregulation

RBP activity is governed not only by genetic alterations or changes in expression, but also by dynamic interactions with the molecular environment [103, 104, 105]. Noncoding RNAs, metabolites, cofactors and other RBPs can regulate RBP function through competitive or cooperative mechanisms, influencing target recognition, binding affinity and post‐transcriptional output. When these regulatory interactions are disrupted, RBP‐centered networks may become rewired to support oncogenic programs. Therefore, delineating the dynamic interactome of RBPs is essential for understanding how their activity is regulated under physiological conditions and how its dysregulation contributes to cancer progression.

### 3.1 Competitive Regulation of RBP Function

**FIGURE 4:**
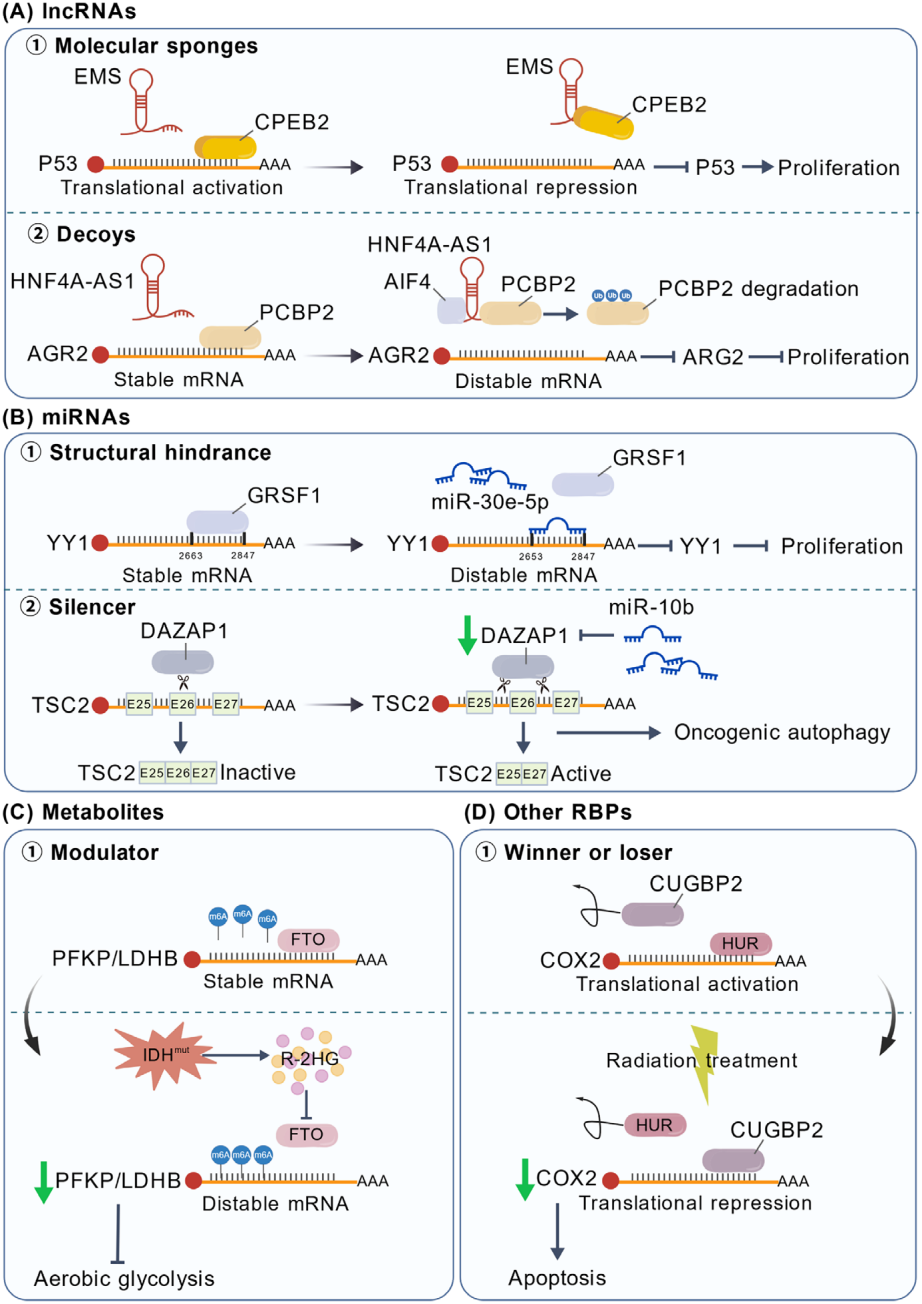
Competitive networks regulating RBPs. Competitive RBP networks involve (A) lncRNAs, (B) miRNAs, (C) metabolites, and (D) other RBPs. (A) LncRNAs suppress RBP function mainly through sequestration or degradation. EMS acts as a molecular sponge to sequester CPEB2 from its target transcripts, inhibiting P53 translation and promoting tumor proliferation, whereas HNF4A‐AS1 promotes ubiquitin‐mediated PCBP2 degradation, reducing ARG2 mRNA stability and suppressing tumor progression. (B) miRNAs inhibit RBPs through structural competition or direct silencing. miR‐30e‐5p competes with GRSF1 for overlapping binding sites in the YY1 3′ UTR, blocking GRSF1‐mediated YY1 mRNA stabilization and inhibiting tumor cell proliferation. miR‐10b directly silences DAZAP1, causing aberrant TSC2 splicing and oncogenic autophagy. (C) The oncometabolite R‐2‐hydroxyglutarate (R‐2HG), produced by IDH‐mutant tumors, inhibits FTO, increases global m6A levels, destabilizes glycolytic transcripts such as PFKP and LDHB, and suppresses aerobic glycolysis. (D) HuR and CUGBP2 antagonistically regulate COX‐2 translation. After irradiation, COX‐2 mRNA binding shifts from the activator HuR to the repressor CUGBP2, reducing COX‐2 expression and promoting apoptosis in CRC cells.

Competitive regulation represents a key mechanism by which RBP function is tuned in a context‐dependent manner. Noncoding RNAs, small‐molecule metabolites, and even homologous or heterologous RBPs can act as molecular “decoys” or “competitors,” hijacking or modulating the ability of an RBP to recognize and bind its RNA targets (Figure 4). These competitive interactions can redirect or disrupt normal RBP‐mediated regulation and reshape post‐transcriptional networks in both physiological and pathological conditions, including cancer. Such competitive interactions provide a novel perspective for understanding tumor complexity and heterogeneity, revealing a complex ecosystem where the balance of competing molecules dictates cellular fate.

#### 3.1.1 LncRNAs as Molecular Sponges and Decoys

LncRNAs function as pivotal post‑transcriptional regulators across diverse physiological and pathological contexts, often by competing with RBPs to remodel signaling networks and modulate cellular states [106, 107]. Acting as molecular decoys or sponges, lncRNAs can sequester RBPs and interfere with their normal regulatory activities, leading to altered gene‐expression programs and revealing context‑specific functional vulnerabilities. For instance, lncRNA NEAT1 competes with the tumor suppressor P53‐binding protein 1 (53BP1) for binding to Tudor interacting repair regulator (TIRR), thereby activating DNA repair in a cell‐cycle‐dependent manner [108]. Likewise, NONHSAT136151 perturbs QKI‑mediated post‑transcriptional control by sequestering QKI from its target transcripts [109], and lncRNA EMS disrupts the interaction between CPEB2 and p53 mRNA, thereby reducing p53 translation [110].

Through advanced mechanisms such as liquid–liquid phase separation, noncoding RNA activated by DNA damage (NORAD) partitions pumilio RNA‐binding family member (PUM) proteins into NP bodies to maintain genomic stability, and dysregulation of this process drives BC progression and chemoresistance [111, 112]. Li et al. reported that lncRNA PAXIP1‐AS1 acts as a tumor suppressor by binding and destabilizing poly(A)‐binding protein cytoplasmic 1 (PABPC1), thus inhibiting oncogenic P21‐activated kinase 1 (PAK1) mRNA stabilization and metastasis in GC [113]. Likewise, in HCC, HNF4A‐AS1 promotes poly(RC)‐binding protein 2 (PCBP2) degradation and suppresses anterior gradient 2 (AGR2)‐driven tumor progression [114]. Some lncRNAs orchestrate complex multi‐RBP interactions: SPUD rearranges the binding of antagonistic RBPs calreticulin and CUG triplet repeat RNA‐binding protein 1 (CUGBP1) to enhance p21 translation and cell‐cycle arrest [115], while SNHG6 drives HCC progression by competitively sequestering HNRNPL away from the tumor suppressor SET domain containing 7 (SETD7) mRNA, simultaneously guiding polypyrimidine tract‐binding protein 1 (PTBP1) to degrade leucine zipper transcription factor like 1 (LZTFL1) transcripts [116]. A particularly innovative mechanism involves lncRNA‐encoded peptides. The HOXB‐AS3 locus, for instance, encodes a peptide that competitively inhibits hnRNP A1 by occupying its RGG motif, thereby suppressing metabolic reprogramming [117]. This finding fundamentally expands the functional repertoire of lncRNAs, demonstrating that competitive regulation can be mediated not only by the RNA transcript itself, but also by its small peptide products. Together, these findings illustrate how lncRNAs employ competition through mechanisms such as direct binding, phase separation, peptide inhibition, or coordinated RBP regulation to rewire post‐transcriptional control networks, offering compelling insights for future targeted therapies.

#### 3.1.2 MiRNAs in Competitive Control of Target mRNAs

The intricate interplay between miRNAs and RBPs constitutes a sophisticated layer of post‐transcriptional regulation that is frequently dysregulated in oncogenesis [118, 119, 120]. At the heart of this interaction lies direct competition for shared or adjacent binding sites on target mRNAs [121, 122]. A representative example is the antagonism between the RBP CRD‐BP and miR‐183, which target an overlapping region within the coding sequence of βTrCP1 mRNA; CRD‐BP binding physically obstructs Ago2 recruitment, leading to transcript stabilization [123]. Structural hindrance similarly defines the relationship between HuR and miR‐125b on the p53 3′ UTR, where HuR's occupancy near the miRNA site impedes RISC assembly, alleviates translational repression, and ultimately influences cell fate following DNA damage [124]. Parallel competitive binding is observed between GRSF1 and miR‐30e‐5p within the 2663–2847 region of the YY1 3′ UTR, a regulatory clash that modulates YY1 expression in HCC [125]. Beyond direct competition for identical sites, RBPs can also exert their antagonistic function by binding nearby regulatory elements; for instance, HuR promotes Erb‐B2 receptor tyrosine kinase 2 (ERBB‐2) expression in prostate cancer by binding a U‐rich element adjacent to the miR‐331‐3p target site, thereby sterically hindering the miRNA's repressive function [126].

Beyond spatial competition, a broader regulatory landscape emerges where miRNAs directly target RBPs to enact extensive oncogenic reprogramming. In esophageal squamous cell carcinoma (ESCC), miR‐10b silences the DAZ‐associated protein 1 (DAZAP1), resulting in aberrant tuberous sclerosis complex 2 (TSC2) splicing that generates a constitutively active isoform and drives oncogenic autophagy [127]. MiR‐9 enhances chemosensitivity by directly suppressing the oncogenic RBP eukaryotic translation initiation factor 5A‐2 (EIF5A2), thereby inhibiting EMT in HCC [128].

Notably, the function of tumor‐suppressive miRNAs can themselves be subverted through post‐transcriptional modification. ADAR1‐mediated A‐to‐I editing of miR‐3144‐3p in HCC not only ablates its ability to repress the oncogenic RBP MSI2, but also confers a gain‐of‐function phenotype by enabling suppression of the tumor suppressor SLC38A4 [129]. These interactions can further evolve into self‐reinforcing oncogenic circuits, as illustrated in bladder cancer where RBM24 and miR‐625‐5p engage in a positive feedback loop to sustain a high‐proliferation state [130]. In summary, the relationship between miRNAs and RBPs is a constant tug‐of‐war. They either compete directly for binding sites on mRNA, or miRNAs act to silence the RBPs themselves. When this delicate balance is disrupted, it becomes a powerful driver of cancer progression and survival.

#### 3.1.3 Metabolite‐Mediated Modulation of RBP Activity

To meet the demands of rapid proliferation, cancer cells undergo metabolic reprogramming characterized by the Warburg effect, hypoxia‐driven lactate accumulation, and dysregulated metabolites [131, 132, 133]. Metabolic reprogramming in cancers not only drive malignant progression but also create unique vulnerabilities by regulating the function of RBPs. The fat mass and obesity‐associated protein (FTO) represent a compelling target within this metabolic regulatory network [134, 135]. FTO binds RNA with marked substrate specificity, preferentially engaging m6A‐modified transcripts to regulate oncogenic pathways [136, 137, 138]. Emerging evidence indicates that metabolites can modulate RBP activity in a context‑dependent manner [139, 140]. R‑2‑hydroxyglutarate (R‑2HG), although classically linked to IDH‑mutant tumors, can also exert antileukemic effects in IDH‑wildtype settings by inhibiting methyltransferase‐like 3 (METTL3), increasing global m6A levels, and destabilizing oncogenic transcripts such as MYC [141, 142]. In contrast, the structurally related metabolite D‑2HG acts through an opposite mechanism: by inhibiting the m6A eraser FTO, it raises m6A abundance and increases YTHDF1 binding, thereby enhancing angiopoietin‐like protein 4 (ANGPTL4) translation and promoting metastasis in triple‐negative breast cancer (TNBC). These contrasting effects illustrate how similar metabolites can differentially reshape RBP‑centered regulatory networks depending on cancer context [143].

Distinctly, vitamin E succinate (VES) functions as a molecular glue to promote FTO ubiquitination and degradation. Resulting elevation of m6A on immunoregulatory genes sensitizes tumors to T‐cell‐mediated cytotoxicity, thereby helping overcome immunotherapy resistance [144]. Hypoxia is a key feature of the solid TME and is emerging as a promising area for therapeutic intervention [145, 146]. Mao et al. demonstrated that hypoxia restricts HuR function in ESCC, reducing its binding to ZO‐1, Claudin‐1, and Occludin mRNAs and suppressing their translation, thereby promoting premetastatic niche formation [147]. In glioblastoma, hypoxia disrupts the HuR‐Xin actin binding repeat containing 1 (XIRP1) axis, promoting degradation of the XIRP1 that correlates with disease progression and poor survival [148]. Together, these findings raise a central question: why can inhibition of the same RBP (e.g., FTO) produce opposite consequences in different cancers—tumor‐suppressive in some settings yet prometastatic in others? Addressing this will require mapping how metabolic states and microenvironmental cues reshape the local balance of RBPs and the transcript‐specific dependencies they create.

#### 3.1.4 Competition Among RBPs for Shared RNA Targets

The spatial proximity of distinct RBP‐binding sites on mRNAs enables competitive interactions that exquisitely regulate gene expression [149]. Such competition arises when two RBPs bind the same mRNA but elicit opposing effects—for instance, one RBP promotes exon retention during splicing while another induces ES, or one stabilizes the mRNA while another accelerates its degradation [150, 151, 152]. These interactions are mediated by two primary mechanisms: direct steric hindrance, where overlapping binding sites physically block one RBP's access to its target sequence, and structural rearrangements in the mRNA, where binding by one RBP alters its secondary structure to create a conformation incompatible with another RBP's recognition motif [6]. Such competitive regulation is not an isolated phenomenon but widely manifests across multiple layers of RNA metabolism. For example, in splicing regulation, the mutual exclusion of hnRNPC and U2AF65 at Alu‐derived splice sites prevents aberrant exonization and preserves transcript integrity [153]. Conversely, the evolutionary conserved antagonism between CELF and RBFOX families fine‐tunes developmental and disease‐specific splicing outcomes by competing for overlapping binding motifs [154]. Translational regulation offers another lens: METTL16 sequesters eIF4E2, blocking canonical eIF4E‐mediated initiation while boosting oncogenic translation of stress‐responsive transcripts [155].

Dynamic balance between stabilizing and destabilizing RBPs further illustrates competitive regulation. The HuR and AUF1 engage in a tug‐of‐war at AU‐rich elements (AREs) of transcripts like TNF‐α, where HuR stabilizes proinflammatory signals while AUF1 accelerates decay—a balance dynamically modulated by signaling pathways [156]. ADAR2 stabilizes Cat2 nuclear RNA (Ctn RNA) by binding its 3′ UTR, preventing HuR/PARN‐mediated degradation and modulating RNA decay pathways critical for tumor cell survival [157]. HuR and CUGBP elav‐like family member 2 (CUGBP2) are structural homologs that stabilize COX‐2 mRNA yet are functional antagonists in translation. Upon irradiation, COX‐2 mRNA binding in CRC cells switches from the activator HuR to the repressor CUGBP2 [158]. Remarkably, even noncanonical RBPs exploit these competitive strategies. Nuclear pyruvate kinase m2 (PKM2), a metabolic enzyme better known for its role in glycolysis, binds RNA G‐quadruplexes (rG4s) to physically displace repressive RBPs like HNRNPF. This act activates prometastatic transcripts, revealing a clever crosstalk between metabolism and ribonucleoprotein complexes [159]. And in miRNA‐mediated silencing, family with sequence similarity 120 member A (FAM120A) and tripartite motif‐containing protein 71 (TRIM71) compete with AGO2 for target access or for the central scaffold protein trinucleotide repeat containing 6 (TNRC6), creating a tunable silencing network that drives oncogenic programs [160]. These studies collectively demonstrate that RBP competition is not a molecular quirk but an integrated system. By establishing a “molecular democracy” where multiple RBPs contest regulatory outcomes, cells achieve context‐dependent control over proliferation, stress responses, and disease progression.

#### 3.1.5 Other Competitors With RBPs

While classic RBP competitors—including lncRNAs, miRNAs, metabolites, and fellow RBPs—have been thoroughly reviewed, emerging evidence reveals novel antagonistic relationship that redefine post‐transcriptional control mechanisms. For example, in TNBC, the exosome complex surprisingly antagonizes the RBP Jab1 by competing for binding to homologous recombination repair (HRR)‐associated mRNAs, thereby destabilizing Jab1‐mediated mRNA stabilization and conferring synthetic lethality with PARP inhibitors [161]. The Ewing sarcoma oncoprotein EWSR1::FLI1 not only acts as a transcriptional activator but also promotes mRNA decay [162, 163]. Galvan et al. show that its FLI1 domain directly binds and antagonizes HuR, thereby destabilizing mRNAs and rendering EwS cells hypersensitive to HuR inhibition [164]. Furthermore, Epstein–Barr virus (EBV) transcriptional activator EBNA2 antagonizes the RBP‐J Kappa through a dual mechanism: disrupting its recruitment of corepressor complexes and deploying its intrinsic activation domain to override transcriptional repression [165]. While these studies provide critical insights, significant gaps remain in our mechanistic understanding of RBP antagonism. Furthermore, translating these findings into viable clinical strategies constitutes a long‐term objective, necessitating concerted research efforts.

Collectively, competition serves as a “molecular brake” or “steering wheel,” providing negative regulation, rapid response capability, and fine‐tuning of RBP function. It enhances the plasticity and adaptability of cancer cells, allowing them to navigate diverse selective pressures.

### 3.2 Cooperative Mechanisms Involving RBPs

**FIGURE 5:**
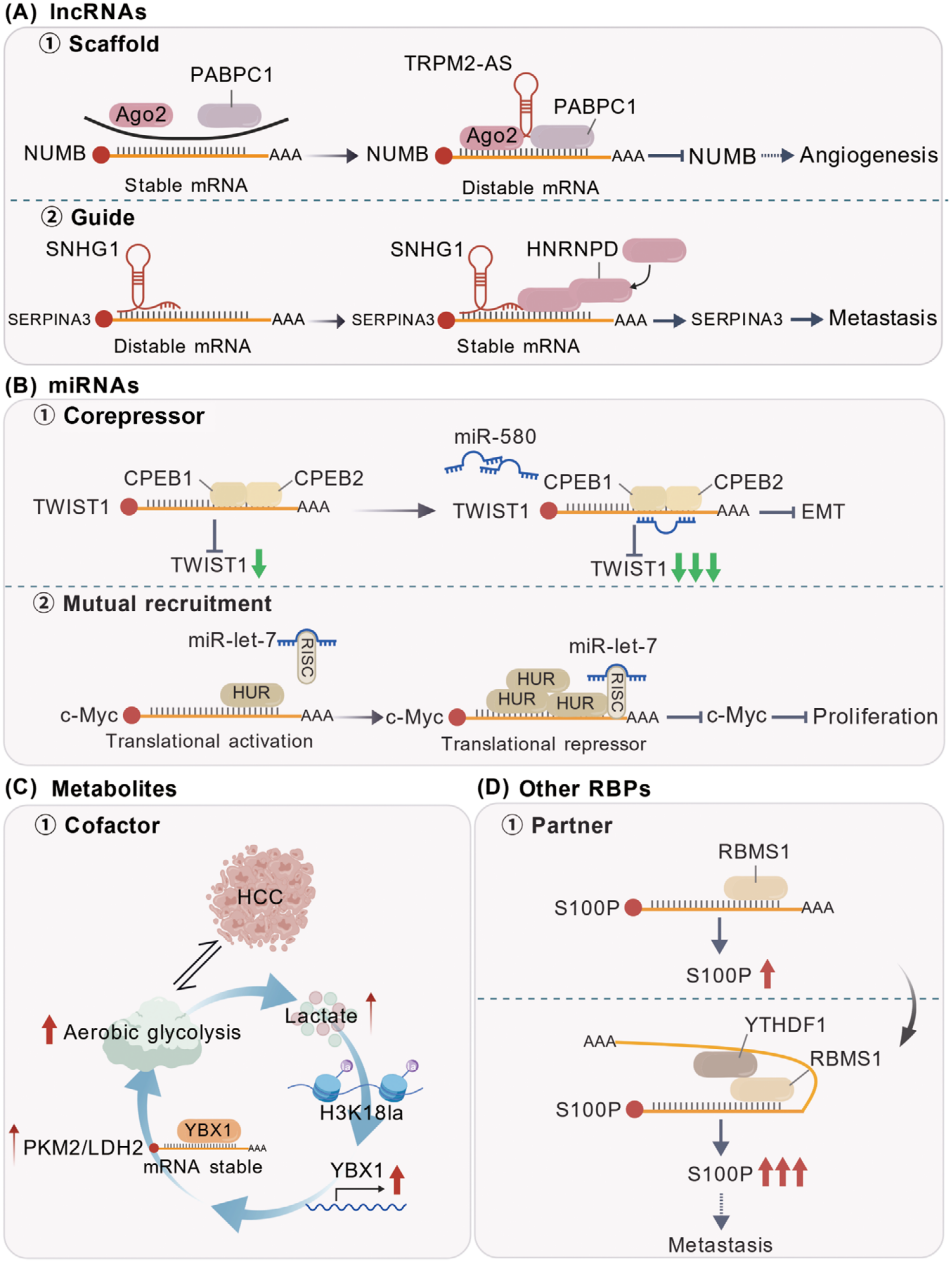
Collaborative networks of RBPs. Collaborative RBP networks involve (A) lncRNAs, (B) miRNAs, (C) metabolites, and (D) other RBPs. (A) LncRNAs enhance RBP function mainly as scaffolds or guides. TRPM2‐AS acts as a scaffold for Ago2 and PABPC1, recruiting them to NUMB mRNA to promote its degradation and angiogenesis. As a guide, SNHG1 recruits HNRNPD to cooperatively stabilize SERPINA3 mRNA and drive metastasis. (B) miRNAs potentiate RBP activity as corepressors or via mutual recruitment. miR‑580 strengthens the repressive effects of CPEB1/2 on TWIST1 in a sequence‑specific, additive manner, thereby inhibiting EMT. HuR recruits the miR‑let‑7‑loaded RISC to c‑Myc mRNA, while miR‑let‑7 enhances HuR binding at distal sites, forming a reinforced repressive complex that suppresses c‑Myc expression. (C) Metabolites act as cofactors. Lactate promotes O‑GlcNAcylation and stabilization of YBX1, leading to histone H3K18 acetylation and enhanced stabilization of PKM2 and LDH2 mRNAs, thereby reinforcing aerobic glycolysis in hepatocellular carcinoma. (D) Other RBPs serve as collaborative partners. RBMS1 interacts with YTHDF1, inducing structural rearrangements in S100P mRNA and increasing its translation, ultimately promoting tumor metastasis.

Previous studies have extensively characterized how RBPs engage in competitive binding with other RBPs, noncoding RNAs, or even small molecules to determine the fate of target RNA. These competitive dynamics have deepened our understanding of RBP‐mediated dysregulation in cancer. However, cancer biology increasingly reveals that regulatory networks are not just battlegrounds of competition but also arenas of collaboration. Certain studies often fail to capture the full complexity of tumor phenotypes, where RBPs frequently achieve their pathogenic effects by forming synergistic alliances with other biomolecules. Beyond competition, RBPs can cooperate through direct physical interactions or indirect shared targeting to amplify signaling, coordinate multistep processes, or adapt to dynamic microenvironments. This section shifts focus from competitive antagonism to cooperative interplay, systematically exploring how RBPs partner with lncRNAs, miRNAs, metabolites, and other RBPs to shape post‐transcriptional programs and influence cancer‐associated phenotypes (Figure 5).

#### 3.2.1 LncRNAs as Scaffolds and Guides for RBP Partnerships

Beyond their roles as competitive inhibitors, lncRNAs frequently function as essential cofactors that potentiate the protumorigenic activities of RBPs [166]. The cooperative paradigm involves lncRNAs acting as molecular scaffolds, guides, or stabilizers to enhance RBP‐mediated regulation of oncogenic pathways. The lncRNA TRPM2‐AS1, itself stabilized by the IGF2BP2 in an m6A‐dependent manner [167], is packaged into exosomes in gallbladder cancer. Upon delivery to endothelial cells, it partners with the RBP PABPC1 to intensify the suppression of the tumor suppressor NUMB, a synergy that potently activates NOTCH1 signaling and drives tumor angiogenesis [168]. Besides, LINC02159 binds to the RBP Aly/ref export factor (ALYREF) and enhances the stability of YAP1 mRNA through m5C modification, leading to activation of Hippo and β‐catenin signaling and promoting NSCLC progression [169]. Yang et al. discovered that lncRNA SNHG1 facilitates metastasis by recruiting HNRNPD to cooperatively stabilize serpin family a member 3 (SERPINA3) mRNA, thereby promoting CRC cell migration and invasion [170]. Likewise, ABHD11‐AS1 interacts with IGF2BP2 to enhance forkhead box protein m1 (FOXM1) mRNA stability, forming a positive feedback loop that promotes CRC progression; meanwhile, ABHD11‐AS1 also acts as a scaffold to facilitate the interaction between IGF2BP2 and the E3 ligase TRIM21, regulating RBP stability in a context‐dependent manner [171].

LncRNAs can also orchestrate oncogenic programs by critically regulating the protein stability of their RBP partners, thereby amplifying downstream signaling outputs. In gallbladder cancer, MNX1‐AS1 recruits ubiquitin specific peptidase 16 (USP16) to deubiquitinate and stabilize IGF2BP3, inhibiting the Hippo pathway and forming an oncogenic positive feedback loop with TEA domain transcription factor 4 (TEAD4) [172]. A unique form of cooperation is seen in ESCC, where SNHG16 forms a complex with EIF4A3 to enhance the stability of RhoU mRNA, contributing to tumor progression [173]. Furthermore, lncRNA AL133467.2 interacts with the RBP zinc finger CCHC‐type containing 4 (ZCCHC4) to suppress DNA damage‐induced apoptosis, conferring chemoresistance in HCC [174]. Notably, whereas the GAS6‐AS1 promote chemoresistance in CRC by recruiting PCBP1 [175], the ELF3‐AS1 conversely recruits RBP ILF2/ILF3 to activate a tumor suppressor [176]. Collectively, these studies suggest that lncRNAs are not merely passive scaffolds, but context‐dependent specificity determinants that redirect RBPs toward distinct RNA substrates or protein partners, thereby reshaping post‐transcriptional programs in either oncogenic or tumor‐suppressive directions. Targeting these specific lncRNA‐RBP partnerships may offer novel therapeutic avenues for cancer treatment.

#### 3.2.2 MiRNA–RBP Functional Cooperation

While miRNAs and RBPs often function competitively, they can also act cooperatively to orchestrate gene expression. One major mode of cooperation involves the direct synergistic repression of a shared mRNA target. For example, miRNAs such as miR‐503, miR‐20a, and miR‐125b collaborate with PUM1 and PUM2 to synergistically suppress the translation of the oncogene E2F transcription factor 3 (E2F3), a pivotal regulator of cell proliferation [177], even though miR‐503 and miR‐20a have also been found to antagonize the function of RBPs in other contexts [81, 178]. Nairismägi et al. reported that miR‐580, CPEB1, and CPEB2 act in an additive and sequence‐specific manner to repress the expression of TWIST1 that promotes EMT [179]. Additionally, a set of tumor‐suppressive miRNAs (miR‐34a, ‐101, ‐128, ‐137, and ‐138) have been found to cooperatively bind the RBP MSI1, thereby inhibiting its oncogenic functions and reducing proliferation in glioblastoma and medulloblastoma cells [180]. Beyond these specific miRNA–RBP interactions, broader dysregulation of ncRNA‑related regulatory pathways has also been implicated in the pathogenesis of medulloblastoma [181].

Another layer of cooperation involves mutually dependent recruitment mechanisms. A sophisticated example is the functional interdependence between HuR and miRNA let‐7: HuR recruits the let‐7‐loaded RISC to the c‐Myc mRNA, while let‐7 enhances HuR's binding affinity at its distal site, forming a reinforced repressor complex that potently suppresses c‐Myc expression [182, 183]. Notably, RBPs can also serve as critical facilitators of miRNA activity by remodeling RNA structure. For instance, upon growth factor stimulation, PUM1 binds to the 3′ UTR of p27, inducing conformational changes that expose previously masked binding sites for miR‐221 and miR‐222. Such a structural switch licenses miRNA‐mediated suppression of p27, thereby promoting cell‐cycle progression [184, 185]. In summary, miRNAs and RBPs do not act in isolation but often function as collaborative partners. Their cooperation, achieved through direct corepression, structural reshaping, or reciprocal recruitment, constitutes a crucial layer of post‐transcriptional regulation and offers promising therapeutic opportunities.

#### 3.2.3 Metabolites as Cofactors in RBP Activation

Some metabolites directly enhance RBP function through post‑translational modifications and as essential cofactors, enabling advanced RNA regulatory activity that drives tumor progression and therapy resistance and also contributes to other disease processes such as metabolic and cardiovascular disorders. Such cooperation creates integrated metabolic‑transcriptomic networks that operate across diverse disease contexts, with cancer representing one prominent example. Lactate, a key glycolytic metabolite, exemplifies this regulatory mechanism through novel post‐translational modifications [186, 187, 188]. In Lenvatinib‐resistant HCC, lactate drives lysine lactylation of IGF2BP3, enhancing its mRNA‐binding capacity to stabilize phosphoenolpyruvate carboxykinase 2 (PCK2) and nuclear factor erythroid 2‐related factor 2 (NRF2) transcripts and thereby conferring drug resistance through enhanced antioxidant defense [189]. Yang et al. found that glycolytic flux promoting P300‐mediated lactylation of Nucleolin at K477, enabling binding to the primary MAP kinase activating death domain (MADD) transcript and orchestrating alternative splicing to promote full‐length MADD translation and ERK activation in intrahepatic cholangiocarcinoma [190]. Beyond lactylation, lactate fuels a feed‐forward loop in HCC by promoting O‐GlcNAcylation and stabilization of YBX1, leading to transcriptional upregulation of P300 that drives histone H3K18 lactylation and further amplifies YBX1 gene expression [191]. Furthermore, Mi et al. also confirmed that YBX1 function is compromised under α‐ketoglutarate (α‐KG) deficiency, impairing the translation of AMPK and thereby sensitizing liver and lung cancer cells to glucose deprivation [192].

Beyond cancer, metabolite‑dependent RBP regulation plays essential roles in nonmalignant diseases. For example, increased glucose flux in cardiac hypertrophy enhances O‑GlcNAcylation of RBPs such as NONO, altering splicing programs that govern cardiomyocyte contractile function [193]. In metabolic disorders, α‑ketoglutarate depletion impairs RBP‑dependent stress‑response pathways, linking TCA‑cycle imbalance to translational control [194].

Metabolic stress conditions like hypoxia actively reshape RBP function through subcellular redistribution. Blanco et al. showed that hypoxic stress induces HuR cytoplasmic translocation, where it stabilizes PIM1 mRNA by binding to AREs, thereby promoting pancreatic cancer cell survival and chemoresistance [195]. Similar hypoxia‑induced HuR relocalization has been documented in ischemic and inflammatory conditions, where HuR‑dependent mRNA stabilization supports tissue adaptation and survival. Notably, the universal methyl donor S‐adenosylmethionine (SAM) serves as both essential cofactor and key regulator for methyltransferase‐type RBPs [196]. Fluctuations in SAM availability directly regulate the catalytic output of RNA‐modifying enzymes, as observed in the METTL3/14 complex where m6A methyltransferase activity intrinsically couples to SAM levels [197, 198]. Altered SAM metabolism is also implicated in metabolic liver disease, cardiovascular dysfunction, and neurodegeneration, indicating that SAM‑sensitive RNA methylation broadly links metabolic state to RNA fate. Zhang et al. demonstrated that the purine synthesis enzyme phosphoribosyl pyrophosphate synthetase 2 (PRPS2), which upregulated in lung adenocarcinoma (LUAD), drives robust ATP production, thereby supporting sustained SAM synthesis [199]. Microbial metabolites like butyric acid counter metabolic stress by upregulating HuR expression and promoting cytoplasmic accumulation, orchestrating a protective gene regulatory program that inactivates AMPK signaling [200, 201]. Thus, metabolites function as biochemical signals that translate the metabolic state of cells into altered RBP activity through cofactor availability, post‑translational modification, and stress‑induced re‑localization. In this way, metabolic rewiring becomes coupled to RNA fate decisions, enabling cells—including tumor, metabolic, and cardiovascular cells—to rapidly adjust mRNA stability, translation, and splicing in response to nutrient fluctuations, hypoxia, inflammation, or therapeutic pressure. This metabolic–RBP coupling may explain why changes in nutrient availability, redox balance, or metabolite pools can create context‑specific RNA‑regulatory dependencies that are not apparent from genomic alterations alone, and why such mechanisms are increasingly recognized across multiple diseases beyond cancer.

#### 3.2.4 Functional Synergy and Collaborative RBP Networks

In the context of RBP biology, cooperativity extends beyond its classical biochemical definition of enhanced binding affinity [202]. Such interplay manifests in various forms: physical interaction between adjacent or spatially proximal RBPs, synergistic functional regulation without direct contact, and cooperative polymerization of homologous RBP monomers on the mRNA target. At the level of translational control, RBMS1 interacts with YTHDF1 to promote the translation of S100P mRNA, thereby driving metastasis in NSCLC, an interaction dependent on the RRM2 motif of RBMS1 and the YTH domain of YTHDF1 [203]. RBM12 also recruits the translation initiation factor eIF4A2 to enhance the ribosome loading and translation efficiency of JAK1 mRNA [204]. Smith et al. reported that a functional unit comprising La‐related protein 1 (LARP1) and the poly(A)‐binding protein PABPC1 is essential for translational repression of specific target mRNAs downstream of the mTOR signaling pathway [205].

In regulating RNA stability, hexokinase domain containing 1 (HKDC1) cooperates with the oncoprotein Ras‐GTPase‐activating protein‐binding protein 1 (G3BP1) to enhance the stability of the PRKDC transcript, a partnership that promotes invasion and chemoresistance in GC [206]. Wang et al. showed that during germ cell development, dead end protein homolog 1 (DND1) cooperates with nanos C2HC‐type zinc finger 3 (NANOS3) to recruit the translational repressor 4E‐T, leading to suppression of SRY‐box transcription factor 4 (SOX4) mRNA translation and precise control over cell fate decisions [207]. The hnRNP‐associated with lethal yellow (RALY) often exerts its oncogenic functions through partnerships with distinct cofactors. In HCC, RALY collaborates with the splicing factor SF3B3 to regulate an metastasis‐associated 1 (MTA1) pre‐mRNA splicing switch, thereby influencing cholesterol metabolism and promoting tumorigenesis [208]. Similarly, our previous work showed that RALY requires a partnership with RBM15b to promote the translation of PLD2 mRNA, a mechanism that drives CRC liver metastasis [209].

Furthermore, cooperation extends to mRNA nucleocytoplasmic transport and translation initiation. Under nitrogen starvation conditions, Npl3 and Pub1 (the mammalian homolog TIA1) work together to promote the cytoplasmic export of ATG1/ULK1 mRNA and its subsequent interaction with the translational machinery, thereby modulating autophagy [210]. The Nocte protein interacts with translation factors eIF3 and Rack1 via its BAT2 domain to cooperatively promote translation reinitiation of mRNAs with long upstream open reading frames (uORFs), such as the glass mRNA [211]. In short, these examples suggest that RBP cooperation creates emergent regulatory properties that are not predictable from individual RBPs alone, enabling context‐specific RNP modules to couple RNA fate decisions with signaling, metabolism, stress adaptation, and cancer‐associated phenotypes.

#### 3.2.5 Other Cooperators With RBPs

In addition to the cooperators discussed above, RBPs participate in broader cooperative networks that further expand their post‐transcriptional regulatory repertoire. A growing body of evidence suggests that circRNAs frequently act as scaffolds or adaptors within these complexes, thereby facilitating cooperative RBP activity in a context‑dependent manner, and contributing to the formation of regulatory circRNA–RBP–mRNA assemblies reported in recent studies [212, 213, 214, 215]. For example, circEXOC6B stabilizes the RBMS1‐HuR complex to suppress metastasis [216]; circMPP6 bridges muscle excess 3A (MEX3A) and processing bodies to drive mRNA degradation [217]; and circRYK competitively sequesters miRNAs while recruiting HuR [218]. Notably, motor neuron and pancreas homeobox 1 (MNX1) drives immune evasion in ESCC by enhancing PD‐L1 expression. However, it operates through a nonclassical mechanism: instead of activating transcription, MNX1 collaborates with the RBP YBX1 to stabilize PD‐L1 mRNA [219].

Furthermore, signals from the TME are integrated into RBP networks, modulating their stability. Inflammatory signals like TNF‐α protect the RBP interleukin enhancer binding factor 3 (ILF3) from degradation, thereby modulating cell death sensitivity [220]. Besides, some collaborations form sophisticated feedback loops. In glioma, the RBP U2 small nuclear RNA auxiliary factor 2 (U2AF65) stabilizes circNCAPG, which then interacts with and stabilizes the transcription factor ras‐responsive element‐binding protein 1 (RREB1). In turn, RREB1 transcriptionally upregulates U2AF65 expression, thereby closing a positive feedback circuit [221]. A similarly intricate loop operates in pancreatic cancer, where the circRNA cALG8 acts as a scaffold for the CDC‐like kinase 1 (CLK1) to phosphorylate the splicing factor serine/arginine‐rich splicing factor 7 (SRSF7). This phosphorylation promotes the generation of a specific ATM kinase variant, which in turn stabilizes the cALG8 transcript itself, thereby instituting a self‐sustaining cycle that drives chemoresistance [222].

Together, RBP interactions are not merely additive, but organizational: they assemble RBPs into higher order regulatory modules that amplify signals, coordinate sequential RNA‐processing events, and convert transient cues into sustained regulatory states. In contrast to competition, cooperation provides the robustness and plasticity that enable cells to adapt to dynamic microenvironmental and stress conditions, with cancer cells representing a particularly well‐studied example.

### 3.3 Pathological Consequences of RBP Network Dysregulation

**FIGURE 6:**
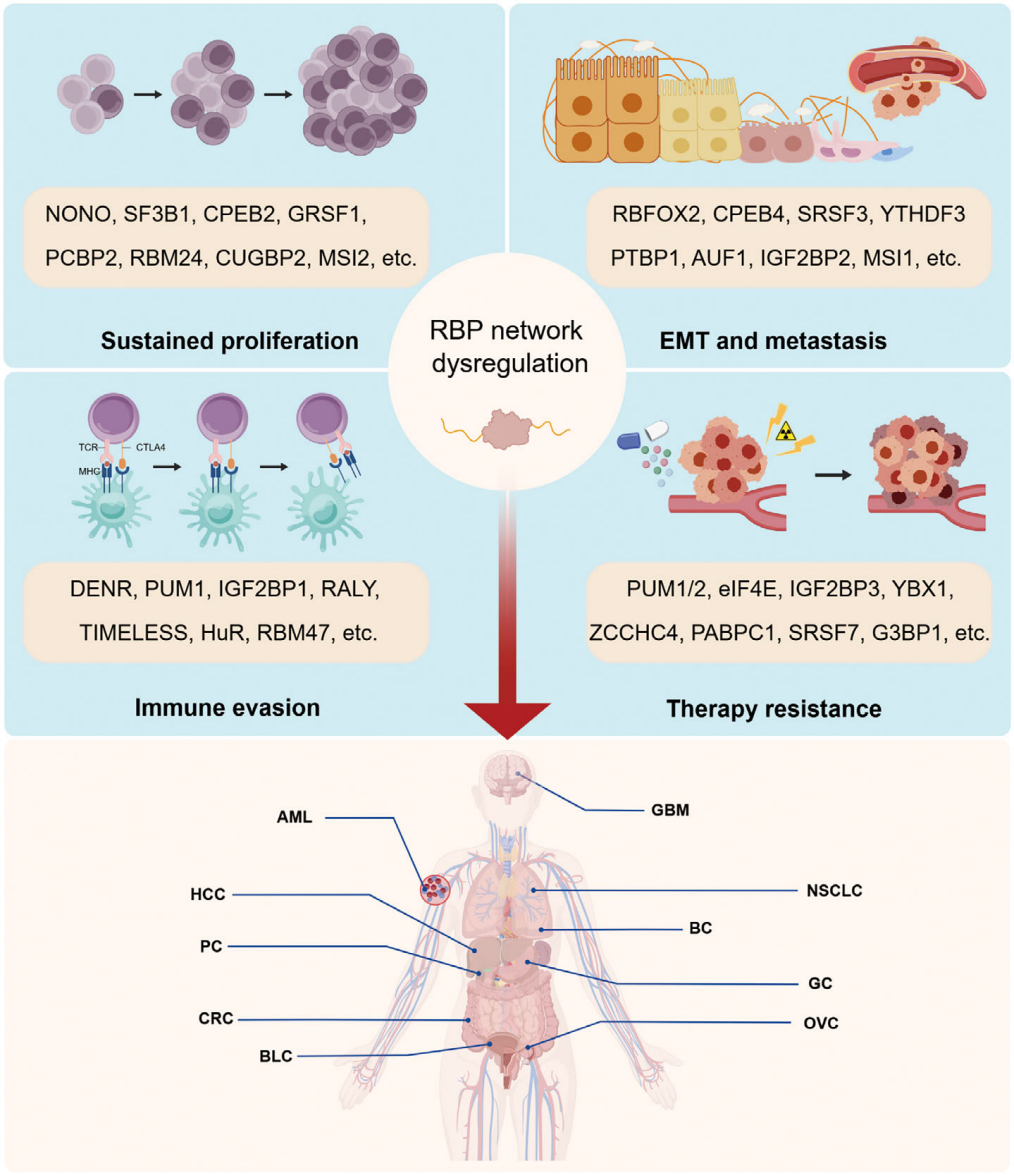
Impact of RBP dysregulation on cancer progression. Representative RBPs contribute to major malignant phenotypes, including sustained proliferation, EMT and metastasis, immune evasion, and therapy resistance. AML, acute myeloid leukemia; BC, breast cancer; BLC, bladder cancer; CRC, colorectal cancer; GBM, glioblastoma; GC, gastric cancer; HCC, hepatocellular carcinoma; NSCLC, non–small‐cell lung cancer; OVC: ovarian cancer; PC, pancreatic cancer.

Although RBP dysregulation contributes to diverse pathological states, the most comprehensive mechanistic insights have emerged from cancer research. Aberrant RBP activity is frequently accompanied by dysregulated expression, stability, translation, or processing of their target mRNAs, which drives hallmark malignant behaviors such as sustained proliferation, metastasis, immune evasion, and therapy resistance (Figure 6). For this reason, our discussion in this section centers on cancer, with selected examples from other diseases included where they illuminate broader principles of RBP‑mediated pathology.

#### 3.3.1 Sustained Proliferation

The regulation of cell proliferation represents the most prevalent function among RBPs with established roles in cancer. One of the most extensively studied examples is the IGF2BP family of RBPs. IGF2BPs are highly conserved across species, and their expression often correlates with poor prognosis [223, 224, 225]. In a renal cell carcinoma (RCC) mouse model, tumor‐specific deletion of IGF2BP1/2/3 each led to a marked reduction in tumor number [226]. Conversely, transgenic overexpression of IGF2BP2 in head and neck squamous cell carcinoma (HNSCC) cells resulted in larger tumors and increased tumor volume in vivo [227]. Overexpression of IGF2BP1 also leads to augmented c‐Myc expression in ovarian cancer and melanoma cells with increased proliferation [228]. IGF2BP2 binds to the mRNAs of EIF4A1, CREB1, and FOXM1, upregulates their expression, and thereby supports proliferation in multiple cancer cell lines [171, 229, 230]. IGF2BP3 is also frequently overexpressed in pancreatic cancer, where it promotes cell proliferation by stabilizing 1,4‐alpha‐glucan branching enzyme 1 (GBE1) and c‐Myc mRNAs [231].

A large number of studies have also implicated HnRNP family in cancer proliferation, which in mammals includes the paralogs hnRNPA1 and hnRNPA2/B1 that are well known for their role in cancer proliferation. Inhibiting hnRNPA1 either with drugs or through gene editing induces apoptosis and cell proliferation [232]. By contrast, hnRNPA1 overexpression develop pancreatic, ovarian cancers, and chronic lymphocytic leukemia, suggesting that in some contexts hnRNPA1 alone is sufficient to induce tumors growth [233, 234]. Knocking out hnRNPA2/B1 significantly inhibited glioma growth in vivo and blocked the G1/S transition in NSCLC cells [235, 236]. Additionally, hnRNPK regulates cell proliferation in multiple contexts. It participates in the post‑transcriptional control of smooth muscle cell growth in response to mitogenic stimulation [237] and also binds to Ras guanyl‐releasing protein 1 (RASGRP1) mRNA to regulate its expression in Jurkat and primary T cells, driving excessive T‑cell expansion and hyperproliferative immune responses [238]. In parallel, hnRNPH1 mediates fibroblast growth factor receptor 2 (FGFR2) alternative splicing, promoting sustained proliferation of vascular smooth muscle cells (VSMCs) and contributing to atherosclerosis [239].

#### 3.3.2 Epithelial–Mesenchymal Transition and Metastasis

EMT is an important pathophysiological event [240]. Increasing evidence shows that EMT promotes cancer cell invasion and migration [241]. Dysregulated expression of several RBPs is involved in EMT in cancer. HuR dysregulation is one of the best‐studied examples. For example, Dong et al. reported that HuR binds to the 3′‐UTR of Snail mRNA, thereby stabilizing it and increasing Snail protein expression in pancreatic cancer cells [242]. This dysregulation leads to a decrease in E‐cadherin expression, which is a well‐known hallmark of EMT. Similar HuR‐mediated regulatory alterations have also been reported in two other related studies. One study showed that this effect is triggered through cooperation with the lncRNA XIST [243], While another study showed that it is induced through cooperation with phosphorylated UDP‐glucose 6‐dehydrogenase (UGDH) [244]. In addition, HuR has been reported to promote EMT in airway epithelial cells from chronic obstructive pulmonary disease patients and in renal epithelial cells from patients with Type 2 diabetes [245, 246].

Beyond the abnormal regulatory networks caused by HuR dysregulation, the cytoplasmic polyadenylation element‐binding protein (CPEB) family has also attracted increasing attention in metastasis. The CPEB family includes CPEB1, CPEB2, CPEB3, and CPEB4. Among them, CPEB1 and CPEB3 are generally considered tumor suppressors that are downregulated in several malignancies [247, 248, 249, 250]. Reduced CPEB1 protein levels are associated with ZO‑1‐related EMT and BC metastasis [62], while CPEB3 suppresses EMT and metastasis in CRC cells [251], suggesting that both CPEB1 and CPEB3 have antimetastatic roles. By contrast, CPEB4 appears to promote EMT and metastasis in several cancer types. CPEB4 accelerates EMT, migration, and invasion in endometrial cancer cells. Its expression is also higher in metastatic lesions [252]. In gallbladder cancer, high CPEB4 expression is closely linked to metastasis and poor prognosis, potentially through enhancing proliferative and migratory capacity [253]. By contrast, CPEB2 appears context dependent: it promotes migration in renal cancer cells [254], yet in HCC it acts in a protective, antimetastatic manner by suppressing EMT [254].

#### 3.3.3 Immune Evasion and the Tumor Microenvironment

RBPs exert multilayered control over immune evasion and remodeling of the TME. Accumulating evidence indicates that multiple RBPs (e.g., DENR, PUM1, and IGF2BP1) promote immune escape by post‐transcriptionally increasing PD‑L1 expression, thereby dampening T‑cell activity and fostering an immunosuppressive milieu microenvironment [255, 256]. Beyond checkpoint regulation, RBPs can also shape key cellular components of the microenvironment and thus sustain immune suppression indirectly. For example, RALY has been reported to drive M2 polarization of tumor‐associated macrophages (TAMs), establishing a liver immunosuppressive niche that favors CRC metastasis [209]; likewise, high TIMELESS expression correlates with reduced T‑cell infiltration and increased M2 macrophage infiltration, promoting LUAD growth [257]. HuR directly binds and stabilizes Foxp3 mRNA, thereby supporting the immunosuppressive function of regulatory T cells (Tregs) [258]. In ovarian cancer, IGF2BP1 suppresses MHC‑I antigen presentation by accelerating IRF1 protein degradation, limiting immune‐cell recruitment and T‑cell activation [259].

Beyond cancer, similar principles extend to infectious and autoimmune contexts, underscoring the broader role of RBPs in immune regulation. In HIV infection, Staufen1 modulates the translation and stability of the viral genomic RNA, enabling HIV to maintain replication and gene expression under host antiviral immune pressure and thereby contributing to immune evasion [260]. In rheumatoid arthritis (RA), ribosomal RNA processing 1 (RRP1) restrains thymidylate synthetase (TYMS) expression and one‑carbon metabolism‐driven inflammation in macrophages, thereby limiting disease severity [261]. Conversely, reduced expression of the RNA‐binding motif protein RBM25 leads to macrophage hyperactivation and tissue inflammatory damage, and has been identified as a major pathogenic factor in RA [262]. Overall, RBPs act as central regulators of immune homeostasis, and their dysregulation can drive immune evasion in cancer as well as pathogenic inflammation in chronic infectious and autoimmune diseases.

#### 3.3.4 Therapy Resistance and Cancer Plasticity

Therapy resistance is increasingly recognized not as a static endpoint, but as a dynamic outcome resulting from cancer cell plasticity, where cancer cells adapt by remodeling transcriptional programs, shifting cellular states, and exploiting microenvironmental cues to withstand therapeutic pressure [263, 264, 265]. At the metabolic level, ferroptosis plasticity supports therapy resistance, as exemplified by MVP‑mediated stabilization of the ferroptosis‑suppressive transcript lipocalin 2 (LCN2), which reduces lipid peroxidation and intracellular Fe^2^^+^ and thereby preserves sorafenib resistance in HCC [266]. Similar RNA‑regulated ferroptosis plasticity occurs beyond cancer; for example, in hypoxic pulmonary hypertension, ALKBH5‑mediated m6A demethylation stabilizes acyl‑CoA synthetase long‑chain family member 4 (ACSL4) mRNA, alters pulmonary artery smooth muscle cell (PASMC) ferroptosis sensitivity [267]. The nucleolar–mitochondrial RNA polymerase I subunit A (POLR1A)/ATF4/TFAM axis that restricts mitophagy‑dependent Fe^2^^+^ release and confers resistance to ferroptosis inducers in pleural mesothelioma [268].

DNA‑damage response plasticity also contributes to therapeutic tolerance. CPEB1 inhibits SIRT1 translation in HCC [269], and in bladder cancer, YTH domain‐containing protein 1 (YTHDC1) stabilizes phosphatase and tensin homolog (PTEN) mRNA in an m6A‑dependent manner to restrain PI3K/AKT signaling; loss of YTHDC1 activates this pathway, enhancing DNA‑damage‑response‑driven cisplatin resistance [270]. In parallel, GBM relies on a distinct RBP dynamic network, where the YBX1 partners with the lncRNA DARS1‑AS1 to stabilize mRNAs encoding G1–S regulators and FOXM1, thereby maintaining homologous recombination capacity, supporting GSC self‑renewal, and sustaining radioresistance [271]. Similarly, RBBP6 overexpression enhances radioresistance in CRC by reshaping cell‑cycle and apoptotic programs [272]. Translational plasticity also enables malignant adaptation, as PABPC1 forms condensates that selectively enhance the translation of leukemogenic mRNAs with complex 5′ UTRs, thereby driving chronic myelogenous leukemia (CML) blast‑crisis progression and tyrosine kinase inhibitor (TKI) resistance [273]. Taken together, these findings point to a unified model: RBPs and their dynamic interactomes shape the post‑transcriptional plasticity that allows cancers to adapt and develop resistance to therapy. At the same time, these RNA‑centered networks expose potential vulnerabilities that could be exploited therapeutically.

## 4 Therapeutic Opportunities Targeting RBPs and Their Regulatory Networks

As discussed above, RBP‐mediated post‐transcriptional regulation contributes to a broad spectrum of cancer hallmarks, including sustained proliferation, metastasis, immune evasion, and therapeutic resistance. These activities rarely arise from isolated molecular events; instead, RBPs operate within dynamic regulatory networks in which cooperative and competitive interactions coordinate RNA processing, stability, localization, and translation to sustain malignant states [274]. This network‐centric perspective challenges the conventional “one drug–one target” model, as functional redundancy, context‐dependent dependencies, and adaptive feedback responses may limit the efficacy of inhibiting individual RBPs. Accordingly, RBP‐directed therapy is increasingly viewed as a strategy to perturb oncogenic RNA‐regulatory networks rather than merely silence single RBP nodes. Therapeutic intervention may therefore focus on vulnerable interfaces, including RBPs, RBP–RBP assemblies, and RBP–cofactor interactions, with the goal of restoring post‐transcriptional homeostasis and attenuating multiple tumor‐promoting phenotypes [275, 276].

With the rapid expansion of RBP research and the continued development of high‐throughput drug screening, RNA‐targeted therapeutics, and targeted protein degradation technologies, several RBP‐focused strategies have entered preclinical and clinical evaluation (Table 1). These include small‑molecule inhibitors that directly block or allosterically remodel RBP–RNA interfaces, or act on cooperating or competing proteins to indirectly modulate RBP function; nucleic acid‐based modalities such as ASOs that provide sequence‑specific control over RBP transcripts or disease‑relevant RNA substrates; and targeted degradation approaches, including PROTACs and molecular glues, that eliminate oncogenic RBPs together with their scaffolding functions and associated interaction partners. In parallel, natural products remain a structurally diverse source of RBP modulators, often exhibiting multitarget activities suitable for reshaping complex tumor‐promoting RNA networks. In the following subsections, we review these therapeutic strategies and discuss their potential applications, current limitations, and future directions for translating RBP‐targeted strategies into cancer therapy.

**TABLE 1:** Representative preclinical and clinical therapeutic strategies targeting RBP‑centered regulatory networks.

Drug type	Drug	Related RBP	Phase	NCT number	Disease	Mechanism	Refs.
ASO	ASO‐cALG8	SRSF7	Preclinical	/	PDAC	Enhance gemcitabine and anti‐PD‐1 sensitivity	[222]
	ASO	PTBP1	Preclinical	/	ESCC	Suppress metastasis	[277]
	ASO	YY1	Preclinical	/	Glioma	Suppress cancer stem cell self‐renewal	[278]
	ASO‐Au‐TAT NPs	HuR	Preclinical	/	NSCLC	Suppress metastasis	[279]
	4EASO	eIF4E	Preclinical	/	NSCLC	Enhance gemcitabine sensitivity	[280]
	RBM20‐ASOs	RBM20	Preclinical	/	HFpEF	Reduce left ventricular stiffness	[281]
	QRL‐201	TDP‐43	I	NCT05633459	ALS	Not mentioned	[282]
	ISIS 396443	SMN2	Preclinical	/	SMA	Save motor neurons	[283]
Small‐molecule drug	Bisantrene	FTO	II	NCT03820908	AML	Inhibits stem‑cell self‑renewal	[284]
	Brequinar	FTO	I, II	NCT03760666	AML	Inhibits stem‑cell self‑renewal	[284]
	Ribavirin	eIF4E	II	NCT00559091	AML	Enhance chemosensitivity	[285]
	Pyrvinium pamoate	HuR	I	NCT05055323	PC	Inhibit proliferation	[286]
	Auranofin	NONO	I, II	NCT01419691	CLL	Inhibit proliferation	[287]
	Tasisulam	RBM39	II	NCT00383292	Melanoma	Suppress metastasis	[288]
	PRI‐724	SAM68	I	NCT01764477	PC	Enhance gemcitabine sensitivity	[289, 290]
	Vorinostat, Trichostatin A	FTO, ALKBH5	I	NCT00801151	CRC	Induce ferroptosis	[291]
	Mithramycin A	MSI‐2	I, II	NCT01610570	Solid tumors	Inhibit invasion	[292]
	E7107	SF3B1	I	NCT00459823	Solid tumors	Inhibit proliferation	[293]
	ANG1005	LRP1	II	NCT01967810	BCBM	Suppress metastasis	[294]
	STM2457	IGF2BP3	Preclinical	/	HCC	Enhance sorafenib and lenvatinib sensitivity	[295]
	STM2457	eIF4E	Preclinical	/	PC	Inhibit proliferation	[296]
	STM2457	YTHDF2	Preclinical	/	HCC	Overcome chemoresistance	[297]
	Zotatifin	eIF4A	I	NCT04632381	COVID‐19	Inhibit SARS‐CoV‐2 replication	[298]
	Pep8	eIF4E	Preclinical	/	BC	Suppress tumorigenesis	[299]
	VE‐821	YY1	Preclinical	/	HCC	Suppress tumorigenesis	[125]
	DKC1125	KSRP	Preclinical	/	CRC	Suppress metastasis	[300]
	CGP57380	eIF4E	Preclinical	/	T‐ALL	Inhibit proliferation	[301]
PROTAC	sc1‐VHLL	FMRP	Preclinical	/	CRC	Enhance immunotherapy efficacy	[302]
	Protac‐LIN28	LIN28	Preclinical	/	CML	Suppress tumorigenesis	[303, 304]
	Protac‐HuR	HuR	Preclinical	/	BC	Inhibit proliferation	[305]
Natural product	Resveratrol	HuR, LIN28A	Preclinical	/	CC	Suppress tumorigenesis	[306, 307, 308]
	d‐borneol	PCBP2	Preclinical	/	NSCLC	Enhance cisplatin sensitivity	[309]
	Dioscin	RBM47	Preclinical	/	GBM	Restore the immune microenvironment	[310]
	Isoliquiritigenin	IGF2BP3	Preclinical	/	NSCLC	Suppress proliferation and invasion	[311]
	24‐epibrassinolide	IGBFP1	Preclinical	/	HCC	Induce apoptosis and energy restriction	[311]
	Harringtonine	METTL3	Preclinical	/	NSCLC	Enhance 5‐FU sensitivity	[312]
	Gossypolone	MSI1	Preclinical	/	CRC	Induce autophagy and apoptosis	[313]
	Neobractatin	MBNL2	Preclinical	/	CRC	Suppress metastasis	[314]
	Silvestrol	eIF4A	Preclinical	/	HEV	Suppresses hepatitis E virus replication	[315]

Abbreviations: ALS, amyotrophic lateral sclerosis; ASO, antisense oligonucleotide; BC, breast cancer; BCBM, brain metastases from breast cancer; CC, cervical cancer; CLL, chronic lymphocytic leukemia; CML, chronic myeloid leukemia; CRC, colorectal cancer; ESCC, esophagus squamous cell carcinoma; GBM, glioblastoma; HCC, hepatocellular carcinoma; HEV, hepatitis E virus; HFpEF, cardiometabolic heart failure; NSCLC, non–small‐cell lung cancer; PDAC, pancreatic ductal adenocarcinoma; SMA, spinal muscular atrophy; T‐ALL, T‐cell acute lymphoblastic leukemia; 5‐FU, 5‐fluorouracil.

### 4.1 Nucleic Acid‐Based Strategies: Antisense Oligonucleotides and RNA‐Targeted Tools

ASOs have emerged as promising precision therapeutics in oncology, capable of silencing disease‐associated genes through Watson–Crick base pairing [316, 317]. ASOs designed to redirect SRSF1‐mediated AURKA 5′ UTR splicing impaired the SRSF1/AURKA/MYC oncogenic axis and inhibited PDAC progression [318]. ASOs targeting H3‐3A mRNA reduced H3.3K27M expression, restored global H3K27 trimethylation, and suppressed tumor growth in preclinical models of diffuse intrinsic pontine glioma (DIPG) [319].

A growing body of evidence is elucidating the sophisticated mechanisms by which ASOs, through targeting upstream noncoding RNAs, indirectly reprogram RBP activity and downstream splicing landscapes [320]. For example, elevated circRNA cALG8 promotes CLK1‐mediated phosphorylation of SRSF7, driving the splicing of an oncogenic ATM variant (ATM203) that underlies gemcitabine resistance and immunosuppression. Critically, therapeutic intervention with a cALG8‐targeting ASO (ASO‐cALG8) effectively disrupts this CLK1–SRSF7 axis and, in combination with gemcitabine and anti‐PD‐1 immunotherapy, significantly reduces tumor burden in preclinical models [222]. Besides, ASO‐directed silencing of lncRNA CTC‐490G23.2 effectively blocks its function as a molecular scaffold for the PTBP1, thereby interrupting the PTBP1‐mediated splicing switch that generates the prometastatic CD44v isoform and promotes EMT in ESCC [277]. Furthermore, in glioma, ASOs targeting the Notch‐induced oncogenic lncRNA TUG1 inhibit its dual functions in sequestering miR‐145 and recruiting polycomb repressive complexes, effectively suppressing cancer stem cell self‐renewal [278].

Outside oncology, ASO‑mediated modulation of RBP‑dependent splicing is also clinically relevant; for example, partial inhibition of RBM20 shifts titin isoforms and improves diastolic function in a cardiometabolic heart failure (HFpEF) mouse model [281]. In neurodegeneration, stathmin 2 (STMN2)‐targeting ASOs restore normal STMN2 splicing and stathmin‑2‐dependent neuronal functions in TDP‑43‐depleted models, and the lead ASO QRL‑201 is now in a Phase I ALS trial [282]. Similarly, in neuromuscular disease, the SMN2‑targeting ASO ISIS 396443 promotes exon 7 inclusion and restores SMN protein levels, supporting its clinical development for spinal muscular atrophy [283].

However, the potent efficacy of ASOs is not solely dependent on target selection; their delivery efficiency presents a critical bottleneck for clinical success [321, 322]. Consequently, innovative delivery systems have been developed to enhance their stability and targeting specificity. For example, gold nanoparticles cofunctionalized with ASOs against the metastasis‐associated lncRNA MALAT1 and a nuclear‐targeting TAT peptide (ASO‐Au‐TAT NPs) significantly improve nuclear internalization, thereby inhibiting tumor cell migration and metastasis in lung cancer models [279]. More ingeniously, another study strategically leveraged the often problematic “protein corona” effect by designing a retinol‐conjugated polyetherimide nanoparticle that actively recruits specific serum proteins. The functionalized nanoparticle thereby achieved precise ASO delivery to activated hepatic stellate cells, demonstrating remarkable efficacy in a liver fibrosis model [323]. In the realm of clinical translation, ASOs demonstrate a unique advantage in targeting traditionally “undruggable” nodes. eIF4E, a common translation initiation factor, interacts with RBPs to synergistically regulate mRNA translational efficiency [324, 325]. The eIF4E‐specific ASO (4EASO) has been demonstrated to effectively suppress eIF4E expression, reduce the translation of multiple oncoproteins like VEGF and c‐Myc, and synergize with gemcitabine in NSCLC models [280]. Although its monotherapy in a Phase I trial for advanced cancer did not yield objective tumor responses, it successfully downregulated elF4E levels in tumor tissues, unequivocally validating its target engagement. These findings strongly suggest that combining ASOs with other treatments constitutes a solid foundation for unlocking their full therapeutic potential, particularly in reprogramming dysregulated post‐transcriptional networks in cancer.

### 4.2 Targeting RBP–RNA Interfaces: Small‐Molecule Inhibitors

The pursuit of small‐molecule drugs represents a complementary approach, focusing on druggable protein–protein and protein–RNA interfaces within the RBP interactome. METTL3 inhibitors provide a representative example of how small molecules can rewire RBP–RNA recognition. STM2457—the first selective METTL3 inhibitor—not only suppresses m6A deposition and leukemogenic transcripts in AML, but has also shown antitumor activity in solid tumors by impairing m6A‐dependent binding of reader RBPs such as IGF2BP3 and YTHDF2. Across hepatocellular, prostate, and drug‑resistant cancers, METTL3 blockade destabilizes oncogenic transcripts and attenuates tumor progression, underscoring the broad therapeutic relevance of targeting m6A‐driven RBP–RNA interfaces [295, 296, 297].

Epigenetic and post‐translational regulators of RBPs have also emerged as actionable nodes. Inhibitors of protein arginine methyltransferase 1 (PRMT1) and protein arginine methyltransferase 5 (PRMT5)—key arginine methyltransferases that modulate RBP activity and RBP–RNA interactions—have demonstrated synergistic antitumor effects in leukemia and pancreatic cancer, and are currently under evaluation in Phase I clinical trials (GSK3368715, NCT03666988; GSK3326595, NCT04676516) [326, 327]. Likewise, HDAC1 inhibition by Vorinostat (SAHA) and Trichostatin A (TSA) has been shown to activate the m6A demethylases FTO and ALKBH5, altering m6A‐dependent RBP engagement with ferroptosis suppressor protein 1 (FSP1) mRNA and sensitizing CRC cells to ferroptosis [291].

Small molecules can also directly disrupt RBP–protein effector interfaces. The synthetic peptide Pep8 mimics a short linear motif of RBM38 and competitively blocks the RBM38–eIF4E interaction, thereby restoring p53 translation and suppressing tumorigenesis [299]. Similarly, small molecules such as 4EGI‑1 and compound 094 inhibit the eIF4E–eIF4G1 interaction, validating the druggability of translation‑associated RBP effector complexes [328, 329]. In parallel, targeting RNA–protein complexes themselves has yielded promising results: inhibitors that block the interaction of the lncRNA HOTAIR with polycomb repressive complex 2 (PRC2) or lysine‐specific demethylase 1 (LSD1) effectively suppress BC metastasis, illustrating the feasibility of pharmacologically modulating oncogenic RNA scaffolds [330, 331].

Notably, Zotatifin (eFT226), an eIF4A‑selective inhibitor, has also been shown to inhibit SARS‑CoV‑2 replication and is being evaluated in a Phase I clinical trial for COVID‑19 (NCT04632381) [298]. In parallel, Tan's group identified Cpd27 as a competitive inhibitor of CUGBP1–RNA binding. Cpd27 blocks CUGBP1‑mediated degradation of IFN‑γ mRNA, thereby preventing hepatic stellate cell activation and improving liver fibrosis in mouse models [332]. Small‑molecule inhibitors therefore offer opportunities for expanding RBP‑based interventions into additional disease areas.

Finally, small molecules can intervene in higher order RBP regulatory circuits. For example, VE‑821 suppresses HCC progression by disrupting the GRSF1–YY1 positive feedback loop, a key axis competitively modulated by miR‑30e‑5p [125]. Together, these studies highlight that small molecules can target RBP–RNA, RBP–protein, and RNA–protein interfaces, as well as upstream epigenetic regulators, thereby remodeling oncogenic post‐transcriptional networks. Nevertheless, the translational potential of these strategies requires further validation in rigorously designed clinical trials.

### 4.3 Protein Degradation Technologies: PROTACs and Molecular Glues for RBPs

Proteolysis‑targeting chimeras (PROTACs) have emerged as a powerful technology for selective protein degradation, with current applications being predominantly concentrated in cancer research. A PROTAC molecule contains three components. These include a ligand that binds to the protein of interest, a ligand that recruits an E3 ubiquitin ligase, and a linker that connects these two ligands in a covalent manner [333]. After the PROTAC binds the target protein and recruits the E3 ligase, the complex initiates ubiquitination and leads to proteasomal degradation [334]. This strategy provides a way to eliminate proteins that are difficult to inhibit with conventional small molecules, including many RBPs.

Recent studies have expanded PROTAC technology toward RBPs. Ghidini et al. developed a nucleic acid‑based design named RNA‑PROTAC [303]. They created a modified heptamer oligonucleotide that mimics the natural RNA element recognized by LIN28A. This oligonucleotide serves as the ligand for the protein of interest. When linked to an E3‑ligase recruiting module, the RNA‑PROTAC binds LIN28A with high specificity and reduces its protein levels in cancer cells. This study demonstrates that synthetic RNA elements can function as effective recognition modules in degradation platforms.

Other nucleic acid‑guided strategies also support the selective degradation of difficult targets. Aptamer‑based PROTACs use structured RNA or DNA sequences that show high affinity for specific proteins [335]. Early studies indicate that aptamers can recruit E3 ligases and promote efficient degradation of oncogenic proteins with RNA‑binding activity. Additional progress in RNA–protein interactome profiling and ligand selection has further enabled the identification of suitable recognition elements for degradation strategies. New approaches also explore the use of molecular glue degraders to regulate RBPs [336, 337]. Molecular glues promote the formation of stable complexes between a target protein and an E3 ligase.

Together, these advances highlight the growing potential of targeted protein degradation technologies for RBPs. RNA‑PROTACs, aptamer‑guided degraders, and other nucleic acid‑based strategies provide promising frameworks for developing next‑generation therapeutics that modulate post‑transcriptional regulatory networks.

### 4.4 Natural Products as Emerging Modulators of the RBP Interactome

Natural products remain a valuable source of chemical scaffolds for cancer drug discovery [338, 339, 340]. Recent studies suggest that they can also modulate post‐transcriptional regulatory networks by targeting noncoding RNA function, RBP‐associated signaling circuits and RNA modification pathways. *Resveratrol*, for example, regulates MALAT1 and NEAT1, two lncRNAs linked to miRNA biogenesis and cancer cell proliferation through RBPs such as HuR and LIN28A [306, 307, 308]. *d‐Borneol* enhances cisplatin sensitivity in NSCLC by promoting NCOA4‐mediated ferritinophagy and reducing PCBP2 expression [309], whereas *dioscin* disrupts an RBM47–NF‐κB feedback loop and remodels the immune microenvironment in GBM [310].

In the context of host–virus interactions, Todt et al. reported that *silvestrol*, a natural product isolated from *Aglaia foveolata*, suppresses hepatitis E virus replication by blocking eIF4A‐dependent viral RNA unwinding and translation [315]. Similarly, sennoside B disrupts the liquid–liquid phase separation of the SARS‐CoV‐2 nucleocapsid protein by inhibiting its RNA‐binding activity, thereby interfering with viral ribonucleoprotein assembly [341].

Natural products have also yielded entry points for targeting the epitranscriptome. *Isoliquiritigenin* (ISL) suppresses NSCLC progression by reducing m6A modification and downregulating IGF2BP3 [342]. *Harringtonine* restores 5‐FU sensitivity in NSCLC by disrupting PARP‐1/METTL3 cooperation and lowering thymidylate synthase expression [312]. *Quercetin* directly binds the m6A demethylase ALKBH5 and has inspired the development of the covalent ALKBH5 inhibitor 18l, which stabilizes tumor‐suppressive mRNAs and promotes differentiation in AML models [343]. Collectively, natural products can act as both modulators of RBP‐centered networks and starting points for more selective epitranscriptomic drug discovery.

### 4.5 Current Preclinical and Clinical Translation, Challenges and Future Directions in RBP‐Targeted Therapy

The therapeutic targeting of RBPs and their regulatory networks is entering a more mature but still early translational phase. A growing number of RBP‐centered therapeutic strategies have advanced from mechanistic studies to preclinical animal models, whereas only a smaller subset has entered clinical evaluation. Current preclinical evidence is mainly concentrated in several therapeutic modalities, including ASO‐based modulation of ncRNA–RBP or splicing networks, small‐molecule regulation of epitranscriptomic and translational programs, targeted degradation of oncogenic RBPs, and natural product‐derived modulation of RBP‐associated pathways. In animal models, ASO‐based approaches have shown efficacy by disrupting oncogenic splicing or ncRNA‐dependent RBP regulatory circuits, such as the cALG8–CLK1–SRSF7 axis in PDAC, PTBP1‐mediated CD44 splicing in ESCC, TUG1‐associated stemness programs in glioma, and MALAT1‐related metastatic networks in lung cancer models [222, 277, 278, 279]. Similarly, preclinical small‐molecule studies targeting METTL3/m6A‐dependent reader interactions, eIF4E‐associated translation complexes, GRSF1–YY1 feedback circuits, KSRP‐containing metastatic complexes, and other RBP‐associated pathways have provided proof‐of‐concept evidence that post‐transcriptional oncogenic programs are pharmacologically tractable [125, 296, 297].

Compared with the expanding preclinical landscape, clinical translation of RBP‐centered therapies remains limited and is largely represented by small molecules, repurposed agents, or indirect modulators of RBP‐associated pathways. For example, ribavirin has been evaluated as an eIF4E‐directed strategy in AML, providing proof‐of‐principle evidence for pharmacological modulation of oncogenic translation [285]. Spliceosome inhibitors such as E7107 have also entered early‐phase clinical testing in patients with advanced solid tumors, supporting the clinical feasibility of targeting RNA‐processing machinery, although their further development has been constrained by toxicity and narrow therapeutic windows [293]. In addition, agents such as bisantrene, brequinar, pyrvinium pamoate, auranofin, tasisulam, PRI‐724, vorinostat, and mithramycin A have been linked to RBP‐related mechanisms, RNA modification pathways, splicing regulation, or translational control in preclinical studies and have been explored in cancer clinical trials [284, 286, 287, 288, 289, 290, 291]. However, for many of these agents, modulation of a specific RBP or RBP‐associated pathway was not necessarily designed as the primary pharmacodynamic endpoint. Therefore, their classification as RBP‐centered therapies should be interpreted with caution. Representative preclinical studies and clinical trials are summarized in Table 1.

Despite these advances, several key issues must be addressed before RBP‐centered strategies can be broadly translated into precision oncology. A major challenge is selectivity. RBPs often regulate large groups of transcripts and participate in multiple cellular processes. Therefore, targeting a central RBP or its associated RNA modification pathway may produce both antitumor effects and unwanted activity in normal tissues. Defining tumor‐specific RBP dependencies, context‐dependent RNA interactomes and cancer‐selective splicing or RNA modification signatures will be essential for improving therapeutic windows.

Each therapeutic modality also presents specific translational considerations. ASOs and other RNA‐targeted tools require improved delivery to solid tumors and extra‐hepatic tissues, together with careful control of immune activation, off‐target hybridization and durable target engagement. Small molecules, PROTACs and molecular glues require highly selective ligands for protein–RNA, protein–protein or degradation interfaces, as well as robust biomarkers to distinguish direct RBP targeting from indirect pathway modulation. Natural products offer useful chemical scaffolds and can illuminate previously unrecognized RBP‐associated circuits, but many require further optimization of pharmacokinetics, bioavailability, target specificity and mechanistic validation before clinical translation.

Future progress will depend on the integration of chemical biology, RNA interactome profiling, structural biology, medicinal chemistry and functional genomics. These approaches can help identify druggable RBP nodes, define tumor‐specific vulnerabilities and guide rational combination therapies. In parallel, biomarkers based on RBP expression, splicing signatures, or downstream transcriptomic changes may support patient stratification and response monitoring in future trials. Overall, although the current translational landscape is still dominated by preclinical studies and early‐phase clinical trials, the growing availability of RNA‐targeted modalities, selective small molecules, and protein degradation technologies provides a rational framework for moving RBP‐centered therapeutic strategies toward precision oncology.

## 5 Conclusion and Future Perspectives

### 5.1 Concluding Remarks

RBPs are indispensable regulators of post‐transcriptional gene expression and act at nearly every stage of RNA metabolism, including pre‐mRNA splicing, RNA processing, stability, localization, translation, and decay. As summarized in this review, their functional versatility is rooted in the structural diversity of canonical and noncanonical RNA‐binding modules, including RRMs, KH domains, dsRBDs, zinc‐finger motifs, RGG/RG‐rich regions, IDRs, and nonclassical RNA‐interacting surfaces. However, RBP function cannot be fully explained by domain composition, RNA‐binding motifs or expression levels alone. Instead, RBPs act as dynamic regulatory hubs whose activity is shaped by RNA substrates, noncoding RNAs, metabolites, cofactors, post‐translational modifications, subcellular localization, and other RBPs. This view connects the structural and functional principles of RBPs with their broader roles in cellular homeostasis, disease progression and therapeutic vulnerability.

A major conceptual message emerging from this review is that RBP biology should be understood at the network level. Competitive mechanisms, including lncRNA‐mediated sequestration, miRNA–RBP antagonism, metabolite‐dependent modulation and competition among RBPs for shared RNA targets, can redirect RNA fate by changing target accessibility, displacing regulatory partners or altering ribonucleoprotein composition [109]. In parallel, cooperative mechanisms allow lncRNAs, miRNAs, metabolites, circRNAs, cofactors, and other RBPs to function as scaffolds, guides, stabilizers, or activators that reinforce specific RNA‐regulatory outputs [189]. These competitive and cooperative mechanisms are not isolated categories but interconnected regulatory modes that together determine the strength, direction and duration of post‐transcriptional responses. Competition provides flexibility and rapid signal responsiveness, whereas cooperation promotes robustness, amplification, and persistence of RNA‐processing programs. The balance between these two modes helps explain why the same RBP can exert context‐dependent, and sometimes opposing, functions across different cell types, disease states and microenvironmental conditions.

Dysregulation of RBP‐centered networks has broad pathological consequences, with cancer representing one of the most extensively studied and therapeutically relevant contexts. In cancers, altered RBP expression, localization, modification, stability, or interaction patterns can reshape oncogenic and tumor‐suppressive transcriptomes, thereby promoting sustained proliferation, EMT and metastasis, immune evasion, and therapy resistance [344, 345, 346]. Importantly, these malignant phenotypes often arise from network‐level rewiring rather than from the isolated dysfunction of a single RBP. This principle is evident in multiple regulatory layers discussed in this review, including mRNA stabilization and decay, spatial RNA localization, translational reprogramming, alternative splicing, m6A‐dependent reader–writer–eraser circuits, ncRNA–RBP interactions and metabolite‐sensitive RBP activity. At the same time, selected examples from neurodegenerative, cardiovascular, metabolic, immune, and infectious diseases indicate that RBP network dysregulation is not unique to cancer, but reflects a broader biological principle linking RNA fate control to disease phenotypes.

### 5.2 Future Perspectives

Based on the current landscape of RBP research, we highlight some key directions that may drive future advances in this field.

#### 5.2.1 Multiscale Mapping of RBP Interaction Networks and Biomolecular Condensates

Most current studies characterize binary RBP–RNA or RBP–RBP interactions, whereas physiological RBP regulation likely occurs within multiplex ribonucleoprotein assemblies and biomolecular condensates. Quantitative parameters such as interaction strength, stoichiometry, binding kinetics, and context‐specific target occupancy remain poorly defined in tumor tissues. Furthermore, phase‐separated condensates—such as NORAD‐nucleated NP bodies that sequester PUM proteins [111] and stress granules assembled by TIA1 [57]—represent higher order organizational principles that cannot be captured by pairwise interaction studies alone. Addressing these challenges will require the integration of CLIP‐based interactome mapping, proximity labeling, single‐cell and spatial transcriptomics, RNA structure probing, proteomics, metabolomics, live‐cell imaging, and CRISPR‐based perturbation screens [347, 348]. In particular, combining spatial transcriptomics with proximity‐dependent biotinylation approaches may enable in situ profiling of RBP–RNA interactions within their native condensate environments, providing spatially resolved maps of post‐transcriptional regulation in heterogeneous tumor tissues.

#### 5.2.2 Computational and AI‐Driven Prediction of Context‐Dependent RBP Vulnerabilities

**FIGURE 7:**
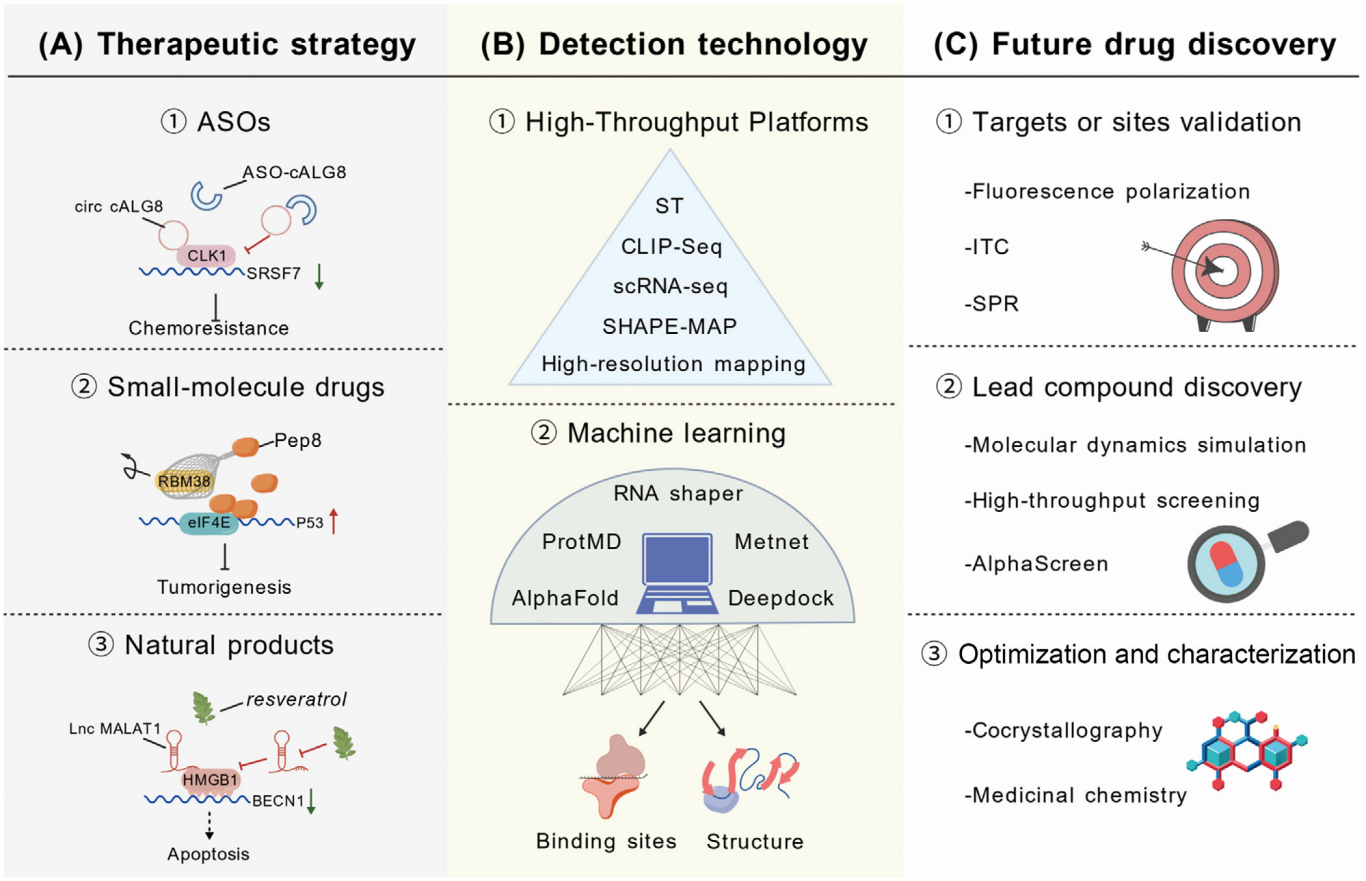
Pharmacological and technological landscape of RBP‐centered networks. (A) Therapeutic approaches targeting RBP networks include antisense oligonucleotides, small molecules, and natural products. (B) High‐throughput and computational technologies, including spatial transcriptomics, scRNA‐seq, CLIP‐seq, SHAPE‐MaP, structural modeling, and molecular docking, enable systematic characterization of RBP expression, RNA structures, binding sites, and interaction networks. (C) Competitive and cooperative RBP networks offer new opportunities for drug discovery, including dual‐function modulators and network‐based combination therapies for preclinical development.

A recurring theme throughout this review is that the same RBP can exert opposing effects in different cancer types or microenvironmental states—for example, FTO inhibition is tumor‐suppressive in AML but may promote metastasis in TNBC through divergent m6A–reader pathways [141, 142, 143], and CPEB2 shows prometastatic activity in renal cancer yet antimetastatic activity in HCC [254]. Predicting such context‐dependent behaviors requires computational frameworks that can integrate multiomics data—including transcriptomic, epitranscriptomic, proteomic, and metabolomic layers—to model dynamic RBP network states. Computational and AI‐driven models, including graph neural networks and large language models trained on RNA–protein interaction datasets, may help predict context‐dependent RBP dependencies, identify network vulnerabilities suitable for therapeutic intervention, and prioritize patient‐specific therapeutic targets [349, 350, 351]. Future efforts should focus on developing machine‐learning frameworks that model how competitive and cooperative interactions collectively determine RBP output, incorporating features such as RNA secondary structure, m6A modification landscapes, metabolite availability, and protein–protein interaction topology (Figure 7). Such approaches could accelerate the identification of actionable RBP nodes and guide the design of rational combination therapies.

#### 5.2.3 Precision Targeting of Disease‐Specific RBP Regulatory Interfaces

This network‐based understanding has important therapeutic implications. Emerging strategies, including ASOs, small‐molecule inhibitors, PROTACs, and natural products, indicate that RBP‐centered regulatory circuits are increasingly druggable. However, a major challenge remains selectivity: because many RBPs regulate hundreds of target transcripts and participate in essential cellular processes, direct inhibition of globally required RBPs may produce unacceptable toxicity. Future therapeutic development should therefore shift from targeting individual RBP proteins toward targeting disease‐specific regulatory interfaces—including tumor‐selective RBP–RNA contacts (e.g., the GRSF1–YY1 axis in HCC [125]), ncRNA–RBP scaffolding interactions (e.g., cALG8–CLK1–SRSF7 in PDAC [222]), metabolite‐sensitive post‐translational modifications (e.g., lactylation of IGF2BP3 [189]), and epitranscriptomic reader–writer–eraser circuits (e.g., METTL3–IGF2BP3/YTHDF2 pathways [295, 296, 297]). Such interface‐directed strategies may preserve essential RBP functions while selectively disrupting oncogenic outputs. Moreover, when combined with chemotherapy, targeted therapy, immunotherapy, radiotherapy, or metabolic intervention, these approaches may help overcome adaptive resistance and tumor plasticity driven by post‐transcriptional rewiring.

#### 5.2.4 Biomarker‐Guided Clinical Translation and Patient Stratification

Despite growing preclinical evidence, clinical translation of RBP‐centered therapies remains at an early stage. A critical bottleneck is the lack of pharmacodynamic biomarkers that capture functional RBP network states rather than static expression levels. Many clinically tested agents—such as ribavirin (eIF4E), bisantrene (FTO), and E7107 (SF3B1)—were not originally developed with RBP modulation as the primary pharmacodynamic endpoint, making it difficult to evaluate true target engagement and predict patient responses. Future clinical trials should incorporate direct target‐engagement assays (e.g., CLIP‐based or proximity‐labeling readouts of RBP occupancy changes), splicing signature panels as surrogate endpoints, and functional biomarkers such as m6A modification profiles, RBP–RNA‐binding ratios, or RBP condensate formation indices. Patient stratification based on RBP network states—rather than single‐gene expression—may identify subpopulations most likely to benefit from network‐targeted interventions. In parallel, the development of companion diagnostics that integrate RBP expression, alternative splicing patterns, and downstream transcriptomic signatures will be essential for advancing RBP‐directed precision oncology from preclinical promise to clinical utility.

In conclusion, RBPs are dynamic regulatory hubs that integrate RNA sequence, structure, modification, noncoding RNA signals, metabolic state, protein partnerships, and microenvironmental cues to determine RNA fate. Decoding the competitive and cooperative logic of RBP‐centered networks, combined with advances in multiomics profiling, AI‐driven modeling, interface‐directed therapeutics, and biomarker‐guided patient stratification, may enable more precise strategies to restore post‐transcriptional homeostasis and exploit RBP‐associated vulnerabilities in precision medicine, particularly in next‐generation precision oncology.

## Author Contributions

**Ling Li and Hui Zhang** conceived the manuscript. **Ling Li** performed the formal analysis and wrote the original draft. **Xiuli Yan, Qing Ji, and Hui Zhang** reviewed and revised the manuscript. All the authors read and approved the final manuscript.

## Funding

This work was supported by grants from the National Natural Science Foundation of China (Nos. 82474125 and 82274297) and the Foundation of Shanghai Municipal Commission of Health and Family Planning (No. 202340073).

## Ethics Statement

The authors have nothing to report.

## Conflicts of Interest

The authors declare no conflicts of interest.

## Supporting information




**Supporting Information File**: mco270938‐sup‐0001‐SuppMat.docx

## Data Availability

The authors have nothing to report.
