# Supplementary material for: RNA‐Binding Proteins: Function, Biological Mechanisms, and Therapeutic Opportunities
Source: MedComm (2020). 2026 Aug 24;7(9):e70938. doi: 10.1002/mco2.70938 (PMC13501125; doi:10.1002/mco2.70938)
Supplement: Supplementary file 1 [file MCO2-7-e70938-s001.docx]

**RNA-binding proteins: function, biological mechanisms, and therapeutic opportunities**

**Running title: Roles of RNA-binding proteins**

Ling Li^1^, Xiuli Yan^2^*, Qing Ji^3^*, Hui Zhang^1^*

^1^Institute of Interdisciplinary Integrative Medicine Research, Shanghai University of Traditional Chinese Medicine, Shanghai 201203, China

^2^Yueyang Hospital of Integrated Traditional Chinese and Western Medicine, Shanghai University of Traditional Chinese Medicine, Shanghai 200437, China

^3^Department of Medical Oncology & Cancer Institute of Integrative Medicine, Shuguang Hospital, Shanghai University of Traditional Chinese Medicine, Shanghai 201203, China.

*Correspondence:

Hui Zhang

zhanghuiman@126.com

Qing Ji

ttt99118@hotmail.com

Xiuli Yan

yxl9999@126.com

**Supplementary Materials**

**1. Search Strategy and Selection Criteria**

This review was designed as a narrative and mechanistically oriented review rather than a formal systematic review or meta-analysis. The literature search aimed to identify representative and high-quality studies describing the biological functions of RNA-binding proteins (RBPs), their regulatory mechanisms, their dysregulation in cancer, and emerging therapeutic strategies targeting RBP-centered regulatory networks.

**2. Databases and information sources**

A comprehensive literature search was performed using the following electronic databases and resources:

PubMed, Web of Science, Scopus, Google Scholar, ClinicalTrials.gov. Additional relevant articles were identified by manually screening the reference lists of key reviews, primary research articles, and highly cited studies. For therapeutic agents and clinical-stage compounds, ClinicalTrials.gov was searched to verify trial phase, cancer type, and available NCT registration numbers when applicable.

The search covered publications from database inception to the most recent update of the manuscript preparation. Priority was given to original research articles, authoritative reviews, and recent studies published in peer-reviewed journals. Seminal older studies were also included when they established fundamental concepts in RBP biology, RNA metabolism, alternative splicing, RNA stability, translation control, RNA localization, or RBP–RNA interaction mechanisms.

**3. Search terms**

**3.1 Example search strings.**

Representative search strategies used in PubMed are shown below. Equivalent terms were adapted for Web of Science, Scopus, and Google Scholar.

3.1.1 General RBP biology and cancer

("RNA-binding protein" OR "RNA binding protein" OR RBP OR "RNA-binding proteins") AND (cancer OR tumor OR tumour OR oncogenesis OR metastasis OR "tumor progression") AND ("post-transcriptional regulation" OR "RNA metabolism" OR "mRNA stability" OR translation OR "alternative splicing" OR "RNA localization")

3.1.2 RBP structure and RNA-binding domains

("RNA-binding protein" OR RBP) AND ("RNA recognition motif" OR RRM OR "KH domain" OR dsRBD OR "zinc finger" OR "RGG motif" OR "intrinsically disordered region" OR IDR)

3.1.3 RBP-mediated RNA stability and decay

("RNA-binding protein" OR RBP OR HuR OR ELAVL1 OR AUF1 OR IGF2BP OR YTHDF2) AND ("mRNA stability" OR "RNA stability" OR "mRNA decay" OR "RNA degradation" OR deadenylation OR decapping) AND (cancer OR tumor OR carcinoma)

3.1.4 RBP-mediated translation regulation

("RNA-binding protein" OR RBP OR eIF4E OR MSI1 OR MSI2 OR HuR OR IGF2BP) AND (translation OR "translation initiation" OR "translational control" OR "translation efficiency") AND (cancer OR tumor OR metastasis OR chemoresistance)

3.1.5 RBP-mediated alternative splicing

("RNA-binding protein" OR RBP OR "splicing factor" OR RBFOX2 OR QKI OR Sam68 OR RBM39 OR SRSF OR HNRNP) AND ("alternative splicing" OR "exon skipping" OR "intron retention" OR "splice site" OR "spliceosome") AND (cancer OR tumor OR leukemia OR carcinoma)

3.1.6 Competitive regulation by lncRNAs, miRNAs, metabolites, and RBPs

("RNA-binding protein" OR RBP) AND (competition OR "competitive binding" OR decoy OR sponge OR "steric hindrance" OR "shared RNA target") AND (lncRNA OR "long non-coding RNA" OR miRNA OR microRNA OR metabolite OR hypoxia OR "RNA-binding protein") AND (cancer OR tumor OR metastasis)

3.1.7 Cooperative regulation and RBP-centered complexes

("RNA-binding protein" OR RBP) AND (cooperation OR cooperative OR synergy OR scaffold OR guide OR cofactor OR "protein-protein interaction" OR "RNP complex") AND (lncRNA OR miRNA OR circRNA OR metabolite OR "post-translational modification") AND (cancer OR tumor OR therapy resistance)

3.1.8 Therapeutic targeting of RBPs

("RNA-binding protein" OR RBP OR "RNA-protein interaction") AND (inhibitor OR "small molecule" OR "antisense oligonucleotide" OR ASO OR PROTAC OR "molecular glue" OR "targeted protein degradation" OR "natural product") AND (cancer OR tumor OR leukemia OR carcinoma OR metastasis OR chemoresistance)

**3.2 Selection criteria**

**3.2.1 Inclusion criteria**

Studies were considered eligible for inclusion if they met one or more of the following criteria:

3.2.1.1 RBP biology and structure

A) Studies describing the classification, structural motifs, RNA-binding domains, intrinsically disordered regions, or biochemical properties of RBPs.

B) Studies identifying canonical or non-canonical RBPs and their RNA-binding capacities.

3.2.1.2 RNA metabolic functions

Studies demonstrating the involvement of RBPs in RNA stability, RNA decay, RNA localization, translation regulation, alternative splicing, RNA modification, stress granule formation, processing body formation, or phase-separated ribonucleoprotein assemblies.

3.2.1.3 Cancer relevance

A) Studies reporting altered expression, mutation, localization, post-translational modification, or functional dysregulation of RBPs in cancer.

B) Studies linking RBPs to cancer-associated phenotypes, including proliferation, EMT, invasion, metastasis, immune evasion, tumor microenvironment remodeling, cancer stemness, metabolic adaptation, chemoresistance, radioresistance, or immunotherapy resistance.

3.2.1.4 Competitive regulatory mechanisms

A) Studies showing that lncRNAs, miRNAs, metabolites, other RBPs, viral proteins, exosome components, or additional molecules competitively modulate RBP binding or function.

B) Studies demonstrating competition for shared RNA targets, overlapping binding sites, adjacent regulatory elements, RNA structural changes, or RBP sequestration.

3.2.1.5 Cooperative regulatory mechanisms

A) Studies showing that RBPs cooperate with lncRNAs, miRNAs, circRNAs, metabolites, cofactors, post-translational modifiers, or other RBPs.

B) Studies describing RBP-containing complexes that regulate RNA fate or cancer-related signaling.

C) Studies demonstrating scaffold, guide, adaptor, stabilizer, cofactor, or feedback-loop functions involving RBPs.

3.2.1.6 Therapeutic targeting

A) Preclinical or clinical studies evaluating small molecules, ASOs, RNA-targeted tools, PROTACs, molecular glues, aptamer-guided degraders, natural products, or combination therapies that directly or indirectly modulate RBP-centered regulatory networks.

B) Clinical trials involving agents with reported relevance to RBP function, RNA modification, translation control, splicing regulation, or RBP-associated pathways.

3.2.1.7 Research type

A) Original research articles, high-quality reviews, mechanistic studies, preclinical studies, translational studies, and clinical trial reports were included.

B) Foundational studies were included when they provided important mechanistic or conceptual background.

**3.2.2 Exclusion criteria**

Studies were excluded if they met any of the following criteria:

3.2.2.1 The article did not focus on RBPs, RNA metabolism, RNA-protein interactions, or RBP-associated regulatory networks.

3.2.2.2 The study discussed cancer biology without mechanistic or functional relevance to RBPs or post-transcriptional regulation.

3.2.2.3 The reported association between an RBP and cancer was purely correlative without sufficient experimental, clinical, or bioinformatic support.

3.2.2.4 The study lacked adequate methodological detail or contained insufficient evidence to support the claimed RBP-related mechanism.

3.2.2.5 The article was a conference abstract, editorial, commentary, news item, or non-peer-reviewed source without sufficient primary data, unless it contained essential information on an ongoing clinical trial.

3.2.2.6 Duplicate publications or overlapping studies from the same group were excluded unless they provided distinct mechanistic, therapeutic, or clinical insights.

3.2.2.7 Non-English articles were generally excluded unless the abstract and available data were directly relevant and interpretable.

3.2.2.8 Studies focused exclusively on non-cancer diseases were excluded unless they provided foundational mechanistic insight into RBP function that was relevant to cancer biology.

**4 Screening and study selection process**

The study selection process consisted of several steps.

First, titles and abstracts were screened to identify studies relevant to RBPs, RNA metabolism, cancer biology, competitive or cooperative regulation, and therapeutic targeting. Articles that did not address RBP-related mechanisms or cancer-associated post-transcriptional regulation were excluded at this stage.

Second, full texts of potentially relevant articles were reviewed. Studies were evaluated for mechanistic clarity, experimental quality, disease relevance, and consistency with the conceptual framework of this review. Particular attention was given to studies that experimentally demonstrated direct or indirect RBP interactions using approaches such as RNA immunoprecipitation, CLIP-seq, eCLIP, RIP-seq, RNA pull-down, mass spectrometry, luciferase reporter assays, RNA stability assays, polysome profiling, ribosome profiling, splicing analysis, RNA modification mapping, proximity labeling, CRISPR screening, or in vivo cancer models.

Third, studies were organized according to the major themes of this review:

A) Structural diversity and fundamental functions of RBPs.

B) RBP-mediated regulation of RNA stability, localization, translation, and alternative splicing.

C) Competitive regulation of RBP function by lncRNAs, miRNAs, metabolites, other RBPs, and additional molecular competitors.

D) Cooperative mechanisms involving RBPs and their molecular partners.

E) Pathological consequences of RBP network dysregulation in cancer.

F) Therapeutic opportunities targeting RBPs and RBP-centered networks.

Finally, selected references were cross-checked to ensure that key mechanistic findings, cancer types, therapeutic agents, and clinical trial information were accurately represented. When multiple studies addressed similar mechanisms, preference was given to recent, comprehensive, mechanistically validated, or clinically relevant studies.

**5 Data extraction and synthesis**

For each selected study, relevant information was extracted, including:

A) RBP name or RBP family.

B) RNA-binding domain or functional motif when available.

C) Cancer type or biological context.

D) RNA substrate or interacting partner.

E) Type of regulatory mechanism.

F) Mode of interaction, including competitive binding, cooperative assembly, scaffolding, sequestration, RNA stabilization, translational regulation, splicing regulation, or RNA modification-dependent regulation.

G) Functional outcome, including proliferation, EMT, metastasis, immune evasion, metabolic adaptation, therapy resistance, or tumor suppression.

H) Therapeutic modality when applicable.

I) Drug or intervention name.

J) Preclinical or clinical status.

K) Clinical trial phase and NCT number when available.

Because this review aimed to integrate mechanistic and therapeutic perspectives rather than quantitatively pool outcomes, no formal risk-of-bias assessment or meta-analysis was performed. Instead, evidence was narratively synthesized to highlight recurring regulatory principles and context-dependent mechanisms across cancer types.

**6 Prioritization of evidence**

Given the broad scope of RBP biology, evidence was prioritized according to the following hierarchy:

A) Mechanistic studies demonstrating direct RBP–RNA, RBP–protein, or RNA–RBP–cofactor interactions.

B) Studies combining molecular assays with functional cancer phenotypes.

C) Studies validated in animal models, patient-derived models, organoids, or clinical specimens.

D) Studies with therapeutic intervention data, including ASOs, small molecules, PROTACs, molecular glues, or natural products.

E) Clinical studies and registered clinical trials involving RBP-associated therapeutic strategies.

F) Seminal studies defining foundational principles of RBP biology, RNA metabolism, or RNA-protein interaction networks.

Particular emphasis was placed on studies that supported the central framework of this review: that RBP function in cancer is not determined solely by RBP abundance or intrinsic RNA-binding capacity, but by dynamic competitive and cooperative interactions within broader post-transcriptional regulatory networks.

**7. Limitations of the search strategy**

Several limitations should be acknowledged. First, because RBP biology is a rapidly expanding field, newly published studies may not have been captured after the final search update. Second, the review was narrative in design and therefore did not apply the strict methodology of a systematic review. Third, the functional annotation of many RBPs remains incomplete, and some proteins classified as non-canonical RBPs may have context-dependent RNA-binding activities that are not fully understood. Fourth, for several therapeutic agents listed in this review, RBP modulation may represent an indirect or recently identified mechanism rather than the original basis for drug development. Therefore, classification of these agents as RBP-centered therapies should be interpreted with caution and requires further pharmacodynamic validation.

Despite these limitations, the search strategy was designed to comprehensively capture major advances in RBP biology, cancer-associated RBP network dysregulation, and emerging therapeutic opportunities targeting RBP-centered regulatory systems.
